# Quantitative Proteomics Identifies Proteins Enriched in Large and Small Extracellular Vesicles

**DOI:** 10.1016/j.mcpro.2022.100273

**Published:** 2022-07-30

**Authors:** Anna Lischnig, Markus Bergqvist, Takahiro Ochiya, Cecilia Lässer

**Affiliations:** 1 Department of Internal Medicine and Clinical Nutrition, Krefting Research Centre, Institute of Medicine at Sahlgrenska Academy, University of Gothenburg, Gothenburg, Sweden; 2 Department of Molecular and Cellular Medicine, Institute of Medical Science, Tokyo Medical University, Tokyo, Japan; 3 Division of Molecular and Cellular Medicine, National Cancer Center Research Institute, Tokyo, Japan

**Keywords:** exosomes, microvesicles, subpopulations, LC-MS/MS, tandem mass tag, ADAM, A Disintegrin and Metalloprotease, ADAMTS, A Disintegrin and Metalloproteinase with ThromboSpondin motifs, BM, MDA-MB-231-luc-BMD2a, CCR4-Not, Carbon Catabolite Repression–Negative on TATA-less, D3H1, MDA-MB-231-luc-D3H1, ER, endoplasmic reticulum, ESCRT, Endosomal Sorting Complex Required for Transport, EV, extracellular vesicle, FBS, fetal bovine serum, hnRNP, heterogeneous nuclear ribonucleoproteins, lEV, large EV, LN, MDA-MB-231-luc-D3H2LN, MV, microvesicle, MVB, multivesicular body, NTA, nanoparticle tracking analysis, sEV, small EV, SNARE, soluble NSF attachment protein receptors, TEAB, triethylammonium bicarbonate

## Abstract

There is a long-held consensus that several proteins are unique to small extracellular vesicles (EVs), such as exosomes. However, recent studies have shown that several of these markers can also be present in other subpopulations of EVs to a similar degree. Furthermore, few markers have been identified as enriched or uniquely present in larger EVs, such as microvesicles. The aim of this study was to address these issues by conducting an in-depth comparison of the proteome of large and small EVs. Large (16,500*g*) and small EVs (118,000*g*) were isolated from three cell lines using a combination of differential ultracentrifugation and a density cushion and quantitative mass spectrometry (tandem mass tag–liquid chromatography–tandem mass spectrometry) was used to identify differently enriched proteins in large and small EVs. In total, 6493 proteins were quantified, with 818 and 1567 proteins significantly enriched in small and large EVs, respectively. Tetraspanins, ADAMs and ESCRT proteins, as well as SNAREs and Rab proteins associated with endosomes were enriched in small EVs compared with large EVs, whereas ribosomal, mitochondrial, and nuclear proteins, as well as proteins involved in cytokinesis, were enriched in large EVs compared with small EVs. However, Flotillin-1 was not differently expressed in large and small EVs. In conclusion, our study shows that the proteome of large and small EVs are substantially dissimilar. We validated several proteins previously suggested to be enriched in either small or large EVs (*e.g.*, ADAM10 and Mitofilin, respectively), and we suggest several additional novel protein markers.

## Highlights

• Quantitative proteomics of small and large extracellular vesicles.
• Tetraspanins, ADAMs, and ESCRT proteins are enriched in small EVs.
• Ribosomal, mitochondrial, and cytokinesis proteins are enriched in large EVs.
• Suggests protein markers for large and small EVs.

## Graphical Abstract

**Figure:**
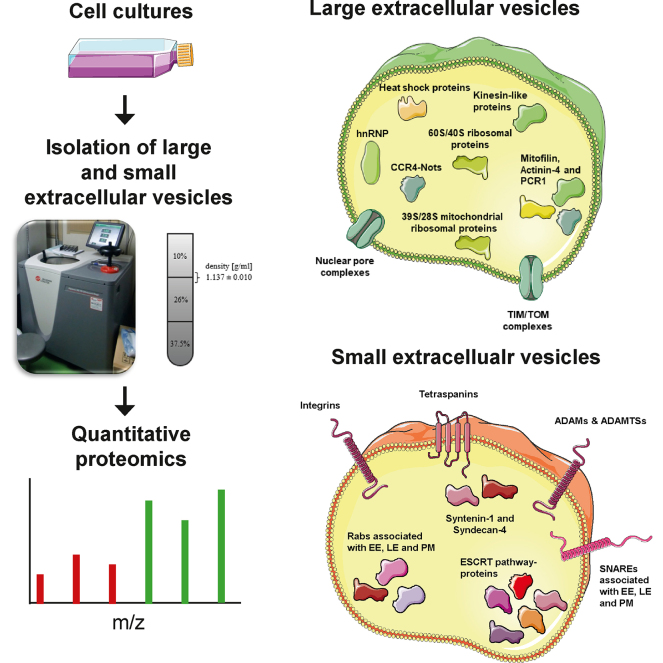

## In Brief

The proteome of large and small extracellular vesicles has been determined with quantitative mass spectrometry. Tetraspanins, ADAMs, and ESCRT proteins, as well as SNAREs and Rab proteins associated with endosomes were enriched in small EVs, whereas ribosomal, mitochondrial, and nuclear proteins, as well as proteins involved in cytokinesis, were enriched in large EVs. Several proteins previously suggested to be enriched in either small or large EVs were validated, and several additional novel protein markers were suggested.

“Extracellular vesicle” (EV) is an umbrella term for nanosized, membrane-enclosed vesicles that are released by cells into the extracellular space. They contain functional RNA, lipids, proteins, and DNA that can be shuttled to recipient cells and change their phenotype (1). EVs have been shown to play a role in a variety of biological processes including inflammation, cancer, homeostasis, and neurodegenerative diseases (1, 2, 3, 4). In addition, EVs and EV-mimetics are studied for their clinical use as biomarkers and therapeutic vehicles for several diseases (3, 4, 5, 6). Recent scientific consensus suggests classification based on size, biogenesis, and sedimentation properties, resulting in three EV subtypes: exosomes that are smaller than 150 nm in diameter, of endocytic origin, and pelleted at >100,000*g* centrifugation. Microvesicles (MVs), which range from 100 to 1000 nm in diameter, are formed when cells shed their plasma membrane and are isolated at 10,000 to 20,000*g*. Lastly, apoptotic bodies that range from 50 nm to 5 μm are shed from dying cells and are usually pelleted at low centrifugation, such as 2000 to 10,000*g* (2, 7, 8, 9).

It was previously believed that all EVs from one cell type induced the same process. However, this simple model has recently been challenged, as apoptotic bodies from dendritic cells induce a T2 response in T cells with the secretion of IL-13, while microvesicles and exosomes from the same cells induce a T1 response with the secretion of IFN-γ (10). Furthermore, in the majority of studies, proteomic analysis has been performed on only one subpopulation of EVs (usually sEVs), resulting in the belief that several proteins were unique for a specific subtype. However, recent studies have shown that several of these markers can also be present in other subpopulations of EVs (11, 12, 13). Furthermore, few markers have been identified as uniquely present in larger EVs (13), as they have been the subject of only a few proteomic studies.

Identifying markers for subpopulations of EVs is of high importance as it will facilitate the possibility to be able to determine in future studies: (1) which EV subpopulation has been isolated; (2) which subpopulation of EVs are responsible for the observed functions; (3) which subpopulations are the most suitable to utilize for vaccine, drug-delivery, and biomarker-discovery; and (4) the differences in biogenesis, cargo, and uptake for these EV subpopulations. To achieve this, better markers are needed to evaluate which EV subpopulations have been isolated and analyzed. We have previously determined the RNA (12, 14, 15) and DNA cargo (16), as well as the morphology (17) of subpopulations of EVs. Here, we use quantitative proteomics to identify differently enriched protein markers for an in-depth comparison of the proteome of subpopulations of EVs.

In this paper, vesicles enriched at 16,500*g* will be referred to as large EVs (lEVs) and vesicles enriched at 118,000*g* will be referred to as small EVs (sEVs), as we do not know their biogenesis. For simplicity, when we refer to published work by others we will use the term sEVs when the authors have used the term “exosomes” or “100K” and lEVs when they have used the term “MVs,” “ectosomes,” or “10K EVs.”

## Experimental Procedures

### Cell Cultures

The breast cancer cell lines MDA-MB-231-luc-D3H1 (hereafter referred to as D3H1), MDA-MB-231-luc-D3H2LN (hereafter LN), and MDA-MB-231-luc-BMD2a (hereafter BM) were used for this project (18, 19). RPMI-1640 medium (HyClone laboratories, Inc) supplemented with 10% EV-depleted fetal bovine serum (FBS), 100 units/ml Penicillin, 100 μg/ml Streptomycin (HyClone), and 2 mM L-Glutamine (HyClone) was used for cell cultures. FBS (Sigma Aldrich) was depleted of EVs by centrifugation at 118,000*g*_avg_ (Type 45 Ti fixed angle rotor, k-factor 178.6; 38,800 rpm, Beckman Coulter) for 18 h at 4 °C using an ultracentrifuge (Optima L-90K Ultracentrifuge, Beckman Coulter). Afterward, the EV-depleted FBS was sterile filtered through a 0.22-μm filter. The cells were seeded at 4.5 × 10^6^/ml, 3.7 × 10^6^/ml, and 3.2 × 10^6^/ml, for D3H1, LN, and BM, respectively. The incubator was humidified and set at 37 °C with 5% CO_2_. The conditioned cell culture media was used to harvest EVs either at 72 or 96 h after cell cultures were split. Conditioned medium from the D3H1 cell cultures was harvested during passages P3 to P12, from LN cell cultures during P3 to P9, and from BM cell cultures during P3 to P10. For each isolation, approximately 600 ml conditioned cell culture medium was harvested.

### Isolation of Extracellular Vesicles With Differential Ultracentrifugation

The primary EV isolation was performed by differential ultracentrifugation, and the following centrifugation steps were all done at 4 °C. First, the conditioned medium from the cells was centrifuged at 300*g* with the SW TTH400 (round bucket) rotor for 10 min to remove cells. The supernatant was then centrifuged at 2000*g* with the SW TTH400 (round bucket) rotor for 20 min to remove large EVs such as apoptotic bodies, as well as cell debris and dead cells. The supernatant was further centrifuged at 16,500*g*_avg_ (Type 45 Ti fixed angle rotor, k-factor 1279.1; 14,500 rpm) for 20 min. The pellet (hereafter referred to as crude lEVs), was resuspended with PBS and stored at −80 °C. Finally, the supernatant was centrifuged at 118,000*g*_avg_ (Type 45 Ti fixed angle rotor, k-factor 178.6; 38,800 rpm) for 2.5 h. This pellet (hereafter referred to as crude sEVs) was also resuspended with PBS and stored at −80 °C.

### Determination of the Density of the Isolated Crude EVs With Density Gradient Centrifugation

Bottom-loaded discontinuous density gradient centrifugations were performed on the crude lEV and sEV pellets from each cell line. The crude pellet had been dissolved in PBS, and PBS was further added to reach a total volume of 1 ml and then mixed with 3 ml 60% iodixanol (Optiprep, Sigma Aldrich), resulting in 4 ml sample/iodixanol solution that had a final concentration of approximately 45%. This solution was loaded onto the bottom of the gradient. The gradient was layered with 1 ml each of 35%, 30%, 28%, 26%, 24%, 22%, 20%, and 10% Iodixanol solution. Next, 200 μl PBS was added on top of the density gradient. The gradient was centrifuged at 180,000*g*_avg_ (SW 41Ti rotor, k-factor 143.9, 38,000 rpm, Beckman Coulter) for 16 h at 4 °C. After centrifugation, the fractions were collected by taking 1 ml at a time from top to bottom. The samples were stored at −80 °C.

### Purification of the Isolated Crude EVs With Density Cushion Centrifugation

The protein measurements, electron microscopy, and Western blots on the density gradient fractions suggested that the majority of the EVs were present in fractions F2 to F4. Therefore, a density cushion including these three fractions was constructed. The crude EV samples had been diluted in PBS, and PBS was now further added to reach a total volume of 1.5 ml and then mixed with 2.5 ml 60% iodixanol. The 4 ml sample/iodixanol solution with an approximate final concentration of 37.5% was loaded at the bottom of the tube. Then 4 ml of 26%, followed by 4 ml 10%, iodixanol solution was layered on top. The cushion was centrifuged at 180,000*g*_avg_ (SW 41Ti rotor, k-factor 143.9, 38,000 rpm) for 2 h at 4 °C. After centrifugation, the interphase between the 10% and 26% iodixanol layer was collected by taking 1 ml.

### Density Measurement

The subsequent density measurement was performed on each fraction of the density gradient and the interphase of the density cushion. Density was determined by measuring the absorbance at 340 nm with a Varioskan LUX microplate reader (SkanIt Software 4.1 for Microplate Readers RE, ver. 4.1.0.43).

### Protein Measurement

The proteins were measured in the crude pellets after ultracentrifugation, in the fractions after the density gradient centrifugation, and in the purified samples after the density cushion centrifugation. The Qubit Protein Assay Kit (Thermo Fisher Scientific) with the Qubit 3.0 Fluorometer was used according to the manufacturer’s instructions.

### Western Blot

The EV samples were thawed and 4× Laemmli buffer (Bio-Rad Laboratories) was added. For the samples that had to be run under reducing conditions, β-mercaptoethanol was added to the 4× Laemmli buffer to a final 1× concentration of 355 mM prior to use. The samples were heated to 95 °C for 5 min and then loaded onto the gels. The samples were separated on Mini-Protean TGX precast 4 to 20% gels (Bio-Rad Laboratories). For volumes and protein amount loaded on the gels see figure legends. The membranes were blocked with a solution of 5% nonfat dry milk in TBST (TBS containing 0.05% Tween-20) or EveryBlot Blocking Buffer (Bio-Rad). The membranes were then incubated with the primary antibodies diluted in 5% nonfat dry milk in TBST overnight at 4 °C. The following antibodies were used: anti-Flotillin 1 (clone EPR6041, ab133497, 1:1000 dilution, Abcam), anti-Calnexin (clone C5C9, 2679, 1:1000 dilution, Cell Signaling Technology), anti-CD63 (Clone H5C6, 556019, 1:1000 dilution, BD Biosciences BD Pharmingen), anti-CD9 (clone MM2/57, 1:1000 dilution, EMD Millipore), anti-CD81 (clone M38, CBL162, 1:1000 dilution, Abcam), anti-syntenin-1 (clone EPR8102, ab133267, 1:1000 dilution, Abcam), anti-ADAM10 (1:500 dilution, clone 163003, MAB1427, R&D System), anti-TOMM20 (1:2000 dilution, clone EPR15581-54, ab186735, Abcam), and anti-RPS7 (1:500 dilution, polyclonal, ab230862, Abcam). To investigate the CD63, CD9, CD81, and ADAM10 expression, the separation was performed under nonreducing conditions. For the other proteins, the separation was performed under reducing conditions. After incubation, the membranes were washed three times with TBST prior to being incubated with the secondary antibodies diluted in 5% nonfat dry milk TBST at room temperature. The following secondary antibodies were used: Donkey anti-rabbit IgG HRP-linked F(ab’)2 fragment (NA9340V) and sheep anti-mouse IgG HRP-linked F(ab’)2 fragment (NA9310V) (dilution 1:5000, GE Healthcare). After incubation, the membranes were washed four times in TBST. The blots were imaged with SuperSignal West Femto Maximum Sensitivity Substrate (Thermo Fisher Scientific) on a ChemiDoc Imaging System (Bio-Rad Laboratories).

### Nanoparticle Tracking Analysis

The samples were diluted in PBS (100- to 1000-fold) directly before the measurement. The camera sensitivity was set to 80, and that of the shutter to 100. Samples were analyzed on a ZetaView PMX instrument (Particle Metrix), and the data were analyzed with ZetaView analysis software version 8.05.11 SP1. The minimum size of the particles was set to 5 nm, the maximum size was set to 1000 nm, and the minimum brightness of the particles was set to 20.

### Transmission Electron Microscopy

Formvar/carbon-coated nickel grids (Ted Pella, Inc) were glow discharged prior to incubation with the samples for 15 min. For volumes and protein amount loaded on the grids see figure legends. Samples were then sequentially fixed with 2% paraformaldehyde and 2.5% glutaraldehyde prior to being negative stained with 2% uranyl acetate. The grids were examined using a Tecnai T12 transmission electron microscope with a Ceta CMOS 16M camera (FEI).

### ExoView

Samples were analyzed with ExoView Plasma Tetraspanin kit on an ExoView R100 instrument (NanoView Biosciences) according to the manufacturer’s instructions. The EV particle concentration in the samples was measured by nanoparticle tracking analysis (NTA) and diluted to 10^8^ particles in 50 μl. This was then further diluted 1:1 using the incubation solution. From each diluted sample, 35 μl was added directly onto the chip and incubated at room temperature for 16 h. The samples were subjected to immunofluorescence staining using fluorescently labeled antibodies (CD9/CD63/CD81, provided in the kit). The chips were then washed, scanned, and analyzed using NanoViewer analysis software version 2.8.10.

### Experimental Design and Statistical Rationale

For tandem mass tag (TMT)–liquid chromatography (LC)–tandem mass spectrometry (MS/MS), lEVs and sEVs were isolated from all three cell lines with ultracentrifugation as described above. Several independent isolations performed on different days were then pooled into three individual pools and loaded onto three independent density cushions per sample type as described above. For each cell line, three density cushion centrifugations with iodixanol were performed per EV type, resulting in three biological replicates. Based on the protein measurements performed on the crude EV pellets after differential ultracentrifugation, the sample material from two crude pellets were loaded onto one cushion for the LN cell line (starting volume 2∗600 ml per biological replicate). As measurements revealed lower amounts of proteins in the lEV and sEV samples of the D3H1 cell line and the BM cell line, the sample material from three crude pellets was loaded onto one cushion for these two cell lines (staring volume 3∗600 ml per biological replicate). This resulted in that three biological replicates were analyzed per EV subtype per cell line, resulting in 18 samples in total (n = 9 for each vesicle subtype in total). The TMT method used allowed comparison of up to ten samples on one set and therefore 9 samples were run on each set. No technical replicates were performed. To compare between sets, a reference pool of all samples was created and was loaded on each set with the same protein amount as for the EV samples. The significance was calculated by paired Student's *t* test on logged values.

### Sample Preparation and Digestion for Mass Spectrometry

A volume corresponding to 45 μg protein was used per sample for all samples, and SDS was added to all samples to reach a final concentration of 2%. The proteomic analysis was performed at The Proteomics Core Facility at Sahlgrenska Academy, Gothenburg University. A reference pool was constructed containing equal amounts of all the samples. The samples and reference pool were digested with trypsin using the suspension trapping (S-Trap, Protifi) spin column digestion method according to the manufacturer instructions. Samples in 2% sodium dodecyl sulfate were reduced with 5 mM dithiothreitol (56 °C, 30 min) and alkylated using 10 mM methyl methanethiosulfonate (room temperature, 20 min). Samples were acidified with phosphoric acid, mixed with S-Trap binding buffer (90% MeOH in 100 mM triethylammonium bicarbonate [TEAB]), transferred to S-Trap micro spin columns, and washed several times with binding buffer. Digestion was performed in 50 mM TEAB, at 37 °C by addition of 1 μg Pierce MS grade Trypsin (Thermo Fisher Scientific) and incubated overnight in a humidified chamber. Peptides were eluted by centrifugation in three steps: (1) 50 mM TEAB, (2) 0.2% formic acid, and (3) 50% acetonitrile, 0.2% formic acid, and the eluates were pooled. The peptides were dried in a sample concentrator, resolved in 50 mM TEAB and, labeled into two sets using TMT 10-plex isobaric mass tagging reagents (Thermo Scientific) according to the manufacturer instructions. The samples within each set were combined and prefractionated into 40 fractions with basic reversed-phase liquid chromatography using a Dionex Ultimate 3000 UPLC system (Thermo Fischer Scientific). Peptide separations were performed using a reversed-phase XBridge BEH C18 column (3.5 μm, 3.0 × 150 mm, Waters Corporation) and a linear gradient from 3% to 40% solvent B over 18 min followed by an increase to 100% B over 5 min and 100% B for 5 min at a flow of 400 μl/min. Solvent A was 10 mM ammonium formate buffer at pH 10.00, and solvent B was 90% acetonitrile, 10% 10 mM ammonium formate at pH 10.00. The fractions were concatenated into 20 fractions, and reconstituted in 3% acetonitrile, 0.2% formic acid.

### NanoLC-MS/MS Analysis and Database Search

Each fraction was analyzed on Orbitrap Fusion Tribrid mass spectrometer (Thermo Fisher Scientific) interfaced with nLC 1200 liquid chromatography system. Peptides were trapped on an Acclaim Pepmap 100 C18 trap column (100 μm × 2 cm, particle size 5 μm, Thermo Fischer Scientific) and separated on an in-house constructed analytical column (350 × 0.075 mm I.D.) packed with 3 μm Reprosil-Pur C18-AQ particles (Dr. Maisch) using a linear gradient from 5% to 35% B over 75 min followed by an increase to 100% B for 5 min, and 100% B for 10 min at a flow of 300 nl/min. Solvent A was 0.2% formic acid in water, and solvent B was 80% acetonitrile in 0.2% formic acid. Precursor ion mass spectra were acquired at 120,000 resolution, scan range 380 to 1380, and maximum injection time 50 ms. MS2 analysis was performed in a data-dependent mode, where the most intense doubly or multiply charged precursors were isolated in the quadrupole with a 0.7 *m/z* isolation window and dynamic exclusion within 10 ppm for 60 s. The isolated precursors were fragmented by collision-induced dissociation at 35% collision energy with the maximum injection time of 50 ms for 3 s (“top speed” setting) and detected in the ion trap, followed by multinotch (simultaneous) isolation of the top five MS2 fragment ions within the *m/z* range 400 to 1200, fragmentation (MS3) by higher-energy collision dissociation at 65% collision energy, and detection in the Orbitrap at 50,000 resolution *m/z* range 100 to 500 and maximum injection time 105 ms.

The data files for each set were merged for identification and relative quantification using Proteome Discoverer version 2.4 (Thermo Fisher Scientific). The search was against Homo Sapiens (Swissprot Database version Mars 2019, 23,443 entries) using Mascot 2.5 (Matrix Science) as a search engine with precursor mass tolerance of 5 ppm and fragment mass tolerance of 0.6 Da. Tryptic peptides were accepted with one missed cleavage, variable modifications of methionine oxidation, and fixed cysteine alkylation; TMT-label modifications of N-terminal and lysine were selected. Percolator was used for peptide spectra matches validation with the strict false discovery rate threshold of 1%. TMT reporter ions were identified with 3 mmu mass tolerance in the MS3 higher-energy collision dissociation spectra, and the TMT reporter abundance values for each sample were normalized within Proteome Discoverer 2.4 on the total peptide amount. Only the quantitative results for the unique peptide sequences with the minimum SPS match % of 65 and the average signal to noise above 10 were taken into account for the protein quantification. A reference sample made from a mix of all the samples was used as denominator and for calculation of the ratios. The quantified proteins were filtered at 5% false discovery rate and grouped by sharing the same sequences to minimize redundancy.

### Statistics and Bioinformatics

Statistical significance was evaluated by ordinary one-way ANOVA and Sidak’s multiple comparison test as a post hoc test in GraphPad Prism (GraphPad Software) for the particle and protein calculations. For these statistical tests, lEVs and sEVs were compared within a cell line. In addition, lEVs and sEVs were compared separately between cell lines. Consequently, the following comparisons were evaluated: LN lEVs *versus* LN sEVs, D3H1 lEVs *versus* D3H1 sEVs, BM lEVs *versus* BM sEVs, and also LN lEVs *versus* D3H1 lEVs, LN lEVs *versus* BM lEVs, D3H1 lEVs *versus* BM lEVs, LN sEVs *versus* D3H1 sEVs, LN sEVs *versus* BM sEVs, and D3H1 sEVs *versus* BM sEVs.

For the proteomic analysis, the significance was calculated by paired Student's *t* test on logged values. The proteins that could be identified by TMT-LC-MS/MS were analyzed using the Database for Annotation, Visualization and Integrated Discovery (DAVID; http://david.abcc.ncifcrf.gov/ [accessed: 08–01–2020]) to determine the cellular components and biological functions of the proteins. Qlucore Omics Explorer (Qlucore) was used for principal component analysis.

## Results

### Large and Small EVs Have the Same Density and Carry Flotillin-1 to a Similar Degree

First, lEVs and sEVs were isolated and characterized from all three breast cancer cell lines used in this study. The LN and BM cell lines released significantly more crude sEVs compared with lEVs, both when protein and particles were measured (Supplemental Fig. S1, *A* and *B*). However, no significant difference was observed for the D3H1 cell line. It has previously been suggested that particle to protein ratio can be used as an estimation of the purity of the isolated EVs (20). While no significant difference was observed, crude lEVs had a higher ratio than the sEVs in all cell lines, which may suggest less contamination of soluble proteins (Supplemental Fig. S1*C*). Western blot showed that flotillin-1 was detected in both the crude lEVs and sEVs, while CD63 and CD81 was exclusively detected or enriched, respectively, in the crude sEVs (Supplemental Fig. S1*D*). The endoplasmic reticulum (ER) protein, calnexin, was mainly detected in the cell lysate, but it also had faint bands in the crude lEVs (Supplemental Fig. S1*D*).

**Fig. 1:**
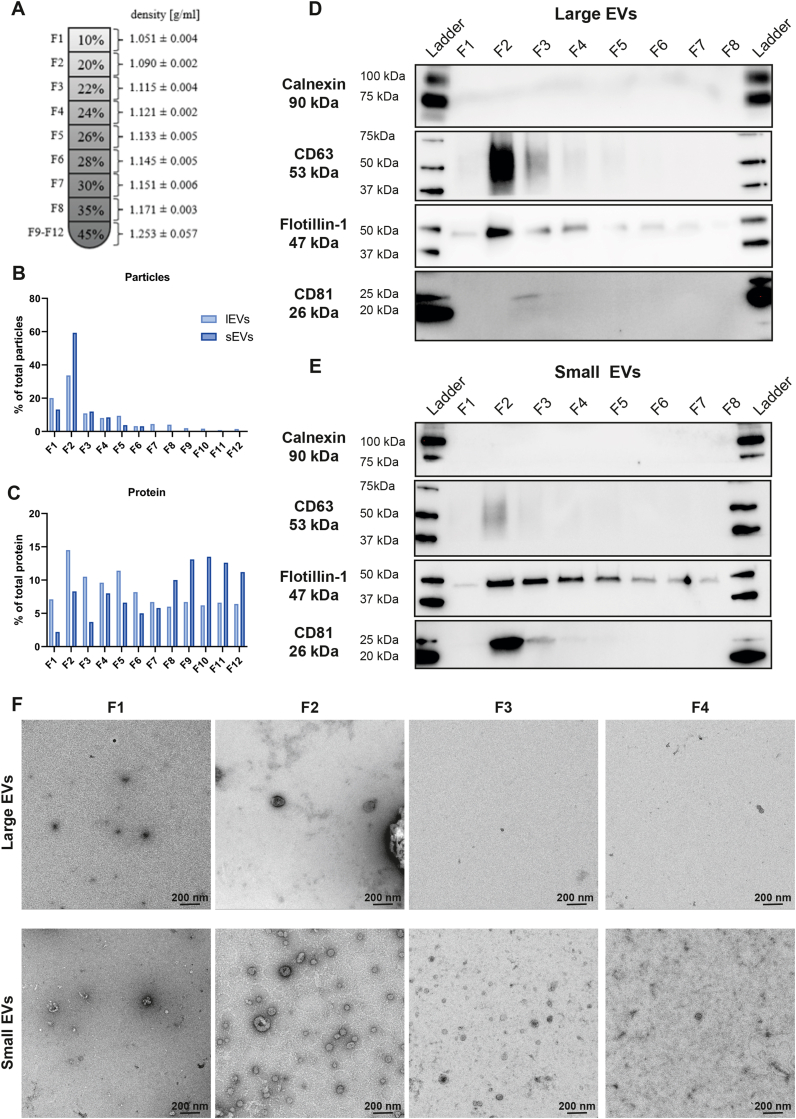
**Flotation on iodixanol gradients shows that both large and small EVs have a buoyant density of 1.1 g/ml.***A*, large and small EVs derived from LN cells were bottom loaded onto iodixanol density gradients. Twelve fractions of 1 ml each were collected from top to bottom from these gradients, and their densities were analyzed by measuring the absorbance at 340 nm. N = 3; result presented as the mean ± SD. *B* and *C*, concentration of particles and proteins in the LN iodixanol gradient fractions determined with Nanoparticle Tracking Analysis (*B*) and Qubit (*C*), respectively. Data presented as the percentage of the total amount of particles or proteins in fractions 1 to 12. N = 1. *D* and *E*, after density flotation and fractionation of LN lEVs and sEVs in high-resolution iodixanol gradients, equal volumes (36 μl; 0.4–8.5 μg protein) of each fraction (fractions 1–8) were loaded onto SDS-PAGE gels. *F*, representative negative staining electron transmission microscopy images of lEVs and sEVs from the LN high-resolution density fractions (fractions 1–4). Thirty microliters (0.6–2.2 μg protein) were loaded onto the grids per each fraction. The scale bars represent 200 nm. EV, extracellular vesicle; lEV, large extracellular vesicle; sEV, small extracellular vesicle.

To determine the density of both the large and small vesicles, crude EV samples were bottom-loaded onto separate density gradients and successive 1-ml fractions were collected from the top (Fig. 1*A*). NTA showed that the majority of particles were found in fraction 2 for both large and small EVs (Fig. 1*B*). Furthermore, a peak was also observed for protein in fraction 2, although this peak was less prominent than for the particles, as the fractions in the bottom also contained a lot of protein, especially for the sEVs (LN EVs; Fig. 1*C*, D3H1 EVs; Supplemental Fig. S2*A*, and BM EVs; Supplemental Fig. S3*A*). Western blot showed that flotillin-1 was present in the majority of all fractions, both for lEVs and sEVs, but with a stronger band in fraction 2 (LN-EVs, Fig. 1, *D* and *E*; D3H2 EVs, Supplemental Fig. S2, *B* and *C*; and BM EVs, Supplemental Fig. S3, *B* and *C*). However, CD63 and CD81 were only present in fraction 2 to 4 and 2 to 3, respectively, but with a stronger band in fraction 2, while calnexin was not detected in any of the fractions. In addition, EM showed that the majority of the vesicles were present in fraction 2 for both lEVs and sEVs (Fig. 1*F*).

These observations indicate that large and small EVs have the same density (1.090–1.121 g/ml) and that both subpopulations carry flotillin-1 to a similar degree. Therefore, neither of these characteristics can be used to differentiate these two subtypes of EVs, which validates previous findings from us and others (11, 13).

### Small Crude EVs Are More Contaminated by Soluble Proteins Than Large Crude EVs

**Fig. 2:**
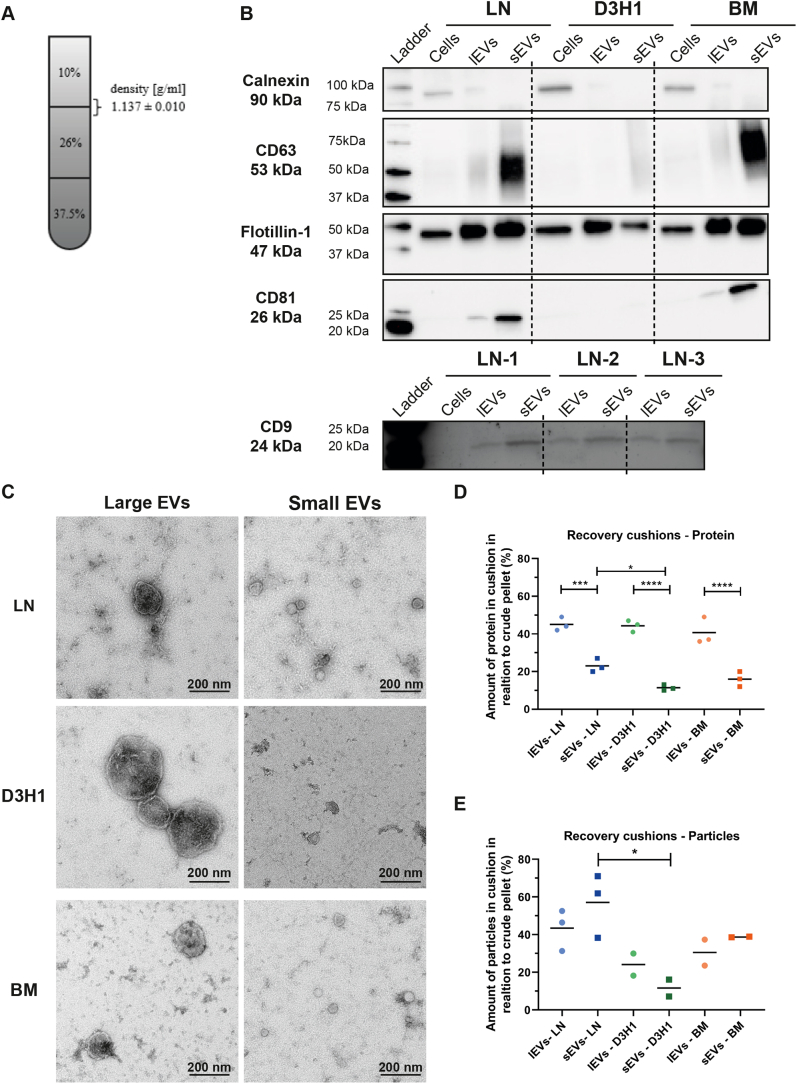
**Flotation on iodixanol cushions shows that both lEVs and sEVs are positive for Flotillin-1 and that sEVs are more contaminated with soluble proteins compared with the lEVs.***A*, large and small EVs derived from LN, D3H1, and BM cells were bottom loaded onto iodixanol cushions. Vesicles were collected in the interphase of 10 and 26%. Densities were analyzed by measuring the absorbance at 340 nm. N = 18, and the result is presented as the mean ± SD. *B*, after density cushions equal amounts of proteins (5.5 μg) of each sample of LN, D3H1, and BM lEVs and sEVs were loaded on SDS-PAGE gels for CD63, CD81, flotilin-1, and calnexin. For CD9 10 μg was loaded for all EV samples and 15 μg for the cell lysate. *C*, representative negative staining electron transmission microscopy images of lEVs and sEVs from LN, D3H1, and BM after iodixanol density cushions. Five micrograms of proteins (13–37 μl) was loaded onto the grids per sample. The scale bars represent 200 nm. *D* and *E*, amount of protein (*D*) and particles (*E*) recovered from the cushions in relation to the amount in the crude pellets that were loaded onto the cushions. N = 2 to 3; ordinary one-way ANOVA and Sidak’s multiple comparison test. ∗*p*-values = 0.05, ∗∗∗*p*-values = 0.001, ∗∗∗∗*p*-values = 0.0001. EV, extracellular vesicle; lEV, large extracellular vesicle; sEV, small extracellular vesicle.

The density gradient results (Fig. 1, Supplemental Figs. S2 and S3) showed that the majority of the particles and vesicle markers were present in fractions 2 to 4. Therefore, we constructed a density cushion isolating these fractions together (Fig. 2*A*). Western blot confirmed our previous results that both lEVs and sEVs were similarly positive for flotillin-1 (Fig. 2*B*). Furthermore, CD63 and CD81 were enriched in sEVs, while calnexin was mainly detected in the cell lysates (Fig. 2*B*). CD9 was not detected in either of the EV subpopulation. This was probably due to that only 5.5 μg protein per sample had been loaded on the gel. The reason for this was that we wanted to load the same amount of proteins for all EV subpopulations from all three cell lines. This resulted in that the low protein concentrations for the BM lEVs and the D3H1 sEVs limited the amount of protein we could load. To evaluate CD9 better, we only used the LN samples as we then could load 10 μg per sample. It was then shown that CD9 was detected in both lEVs and sEVs but was enriched in sEVs (Fig. 2*B*). ExoView showed that both EV subpopulations from all three cell lines were positive for CD81, CD63, and CD9 (Supplemental Fig. S4*A*), indicating that these vesicles do have CD9 on their membrane, although it was below the detection limit for our Western blot setup for two of the cell lines. Electron microscopy validated that vesicles had been isolated and that those in the 16,500*g* pellet were larger than those in the 118,000*g* pellet (Fig. 2*C*). The size difference between the EV subpopulations was further validated with NTA (Supplemental Fig. S4*B*). Recovery was calculated by comparing the yield after the cushion with what was loaded onto the cushion (crude EVs) and showed that protein recovery was significantly higher for lEVs than for sEVs (Fig. 2*D*). However, this was not observed for the recovery of particles (Fig. 2*E*). This is in line with the results from the gradients, where more proteins were observed in the heavier-density fractions (fractions 8–12) for the sEV samples compared with the lEV samples (Fig. 1, *B* and *C*, Supplemental Figs. S2*A* and S3*A*). These findings suggest that crude sEVs are more contaminated with soluble proteins compared with the lEVs, and it is particularly important to purify these on a density gradient or cushion prior to further proteomic analysis.

**Fig. 3:**
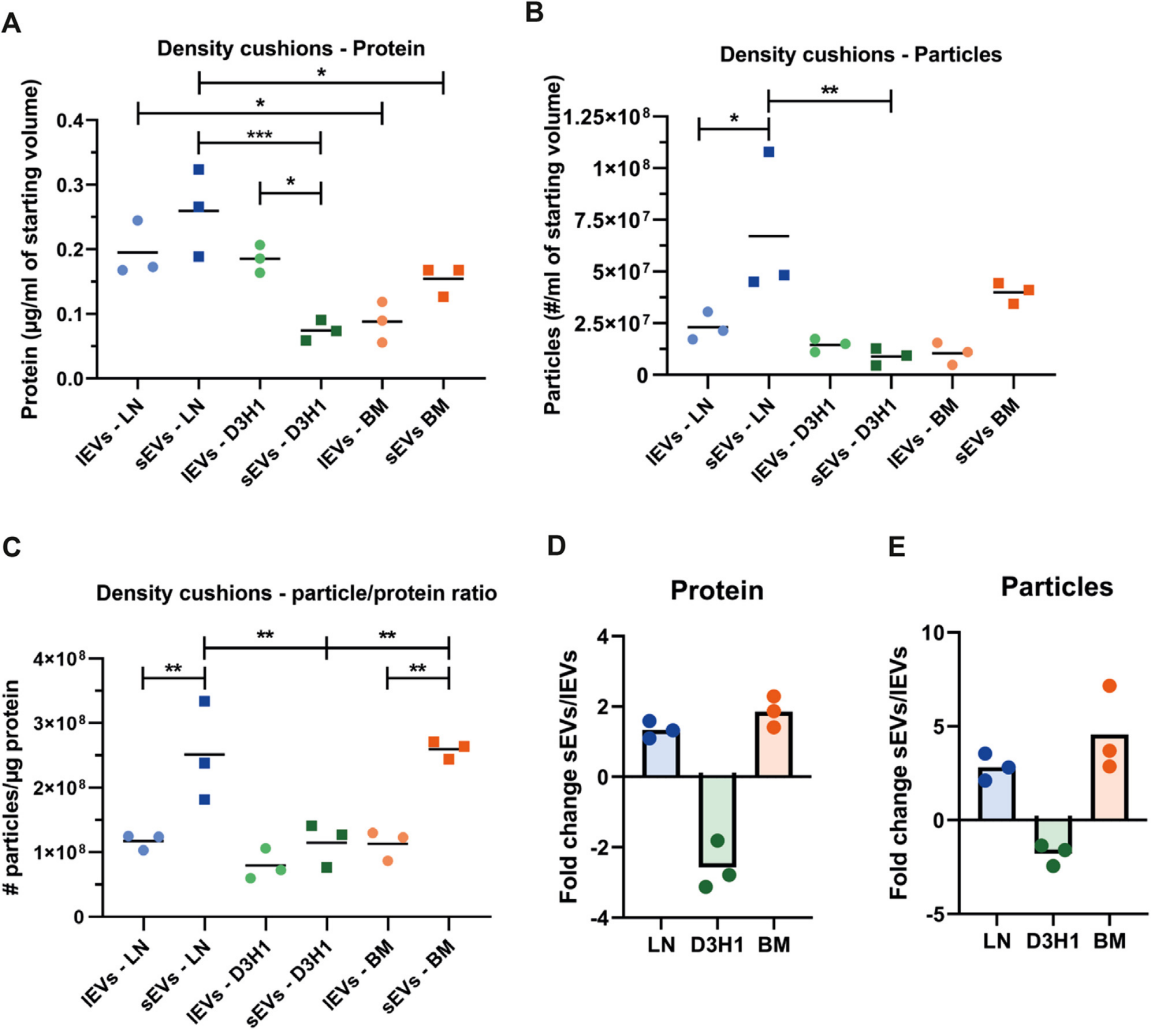
**Highly metastatic breast cancer cells release relatively more sEVs than lEVs, while low metastatic cells released relatively more lEVs.***A* and *B*, amount of proteins (*A*) and particles (*B*) in the cushion enriched and purified EV samples per milliliter starting cell culture media for lEVs and sEVs from all three cell lines. N = 3; ordinary one-way ANOVA and Sidak’s multiple comparison test. ∗*p*-values = 0.05, ∗∗∗*p*-values = 0.001. *C*, particle to protein ratio for all cushion-enriched and -purified extracellular vesicle samples for lEVs and sEVs from all three cell lines. N = 3; ordinary one-way ANOVA and Sidak’s multiple comparison test. ∗∗*p*-values = 0.01. *D* and *E*, sEV to lEV ratio calculated by dividing the absolute protein amount (*D*) and particles (*E*) within each cell line. *C*–*E* is constructed based on the measurements and numbers in *A* and *B*. lEV, large extracellular vesicle; sEV, small extracellular vesicle.

The LN and BM cell lines released relatively more sEVs than lEVs, while the D3H1 cell line released relatively more lEVs than sEVs both when protein and particles were measured (Fig. 3, *A*, *B*, *D* and *E*). In contrast to the crude EVs (Supplemental Fig. S1*C*), significant differences were observed for the particle:protein ratio, which was suggested to measure EV purity (Fig. 3*C*). Interestingly, the particle:protein ratio of sEVs after the cushion was much higher compared with the crude sEVs. In contrast, the ratio for the lEVs remained largely the same, suggesting that the sEVs benefit the most from the purification on the density gradient or cushion.

### Quantitative Proteomics Revealed Significantly Different Proteomes of lEVs and sEVs

**Fig. 4:**
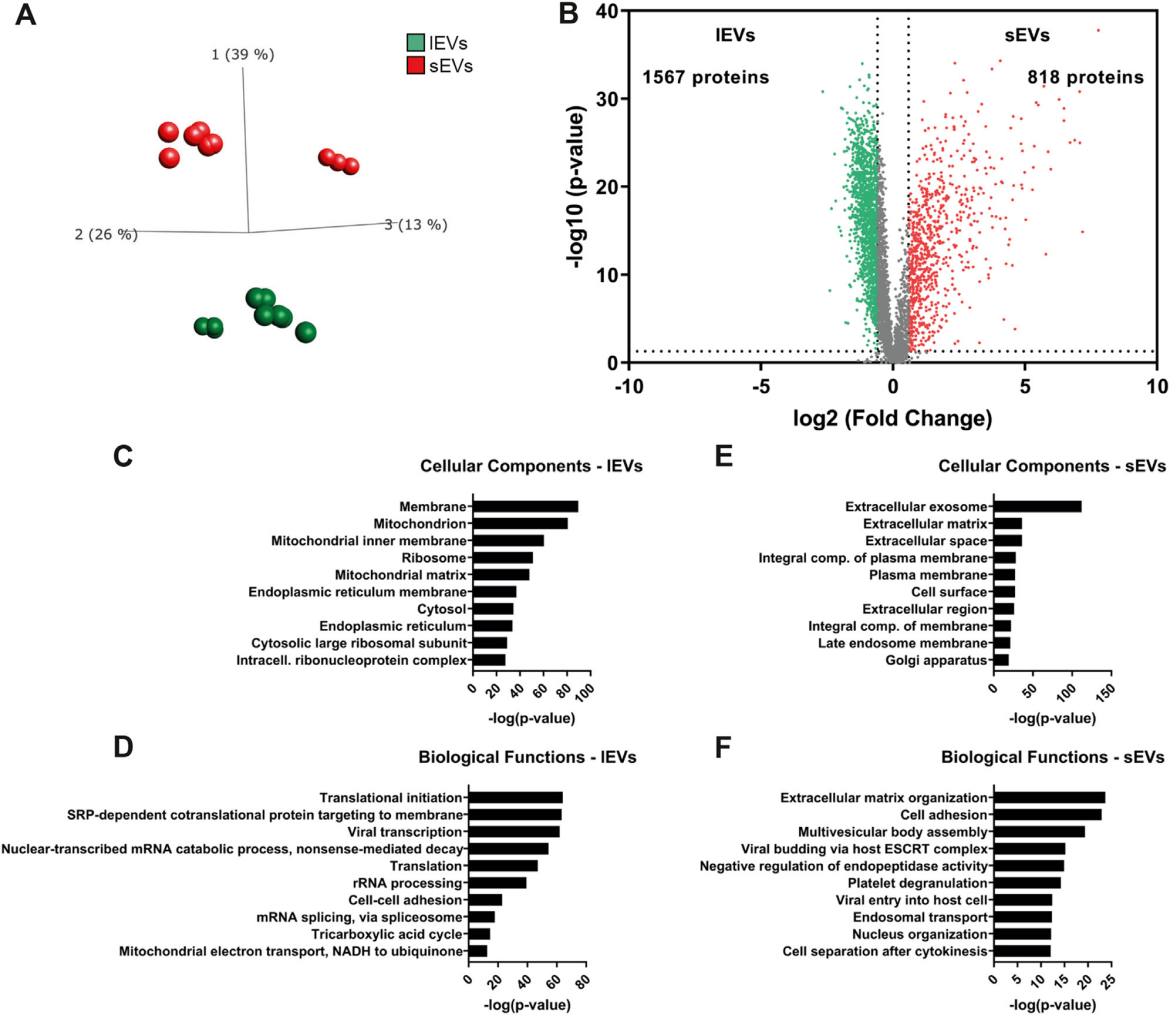
**The proteomes of lEVs and sEVs are substantially dissimilar.** Quantitative proteomics (tandem mass tag) was used to determine the differences in the proteomes of sEVs and lEVs. Three biological replicates (45 μg protein/sample) were used from three different cell lines resulting in N = 9. *A*, principal component analysis illustrating the relationship between sEVs and lEVs derived from the three cell lines. *B*, volcano plot of the proteomes of sEVs and lEVs identified 818 and 1567 proteins, respectively, significantly enriched more than 1.5 fold change. *Dotted lines* indicate cutoffs; 1.3 on the *y*-axis (corresponding to *p* < 0.05) and 0.585 on the *x*-axis (corresponding to fold change >1.5). *C*–*F*, Database for Annotation, Visualization and Integrated Discovery (DAVID) was used to determine the most enriched cellular compartments (*C* and *E*) and biological processes (*D* and *F*) associated with proteins significantly enriched in lEVs (*C* and *D*) and sEVs (*E* and *F*). The ten most enriched terms (based on *p*-value) in each category are displayed. lEV, large extracellular vesicle; sEV, small extracellular vesicle.

We used quantitative proteomics to identify enriched proteins in lEVs and sEVs from three biological replicates from each of the three cell lines. In total, 6493 proteins were quantified, and principal component analysis was performed to visualize the relationship between the different types of isolated EVs (Fig. 4*A*). A clear separation could be seen between lEVs and sEVs by component 1, which represented 39% of the variability. In addition, EVs from the D3H1 cell line were separated from those derived from the LN and BM cell lines by components 2 and 3.

Next, we compared the lEV samples from all three cell lines to the sEV samples (N = 9) and identified 1567 and 818 proteins to be significantly upregulated in lEVs and sEVs, respectively (Fig. 4*B* and Supplemental Table S1). The 1567 proteins enriched in lEVs compared with sEVs were associated with gene ontology (GO) terms indicating that lEVs contained proteins associated with organelles such as the mitochondrion, endoplasmic reticulum, and ribosomes (Fig. 4, *C* and *D*). The 818 proteins enriched in the sEVs on the other hand were associated with gene ontology terms indicating that the sEV proteome was associated with extracellular vesicles and organelles such as multivesicular bodies and late endosomes as well as Golgi apparatus and plasma membrane (Fig. 4, *E* and *F*). This may suggest a different biogenesis for lEVs and sEVs and propose that at least a portion of the sEVs comprise exosomes.

### Small EVs Are Enriched in Tetraspanins, ADAMs, and ESCRT Proteins as Well as SNAREs and Rab Proteins Associated With Endosomes

**Fig. 5:**
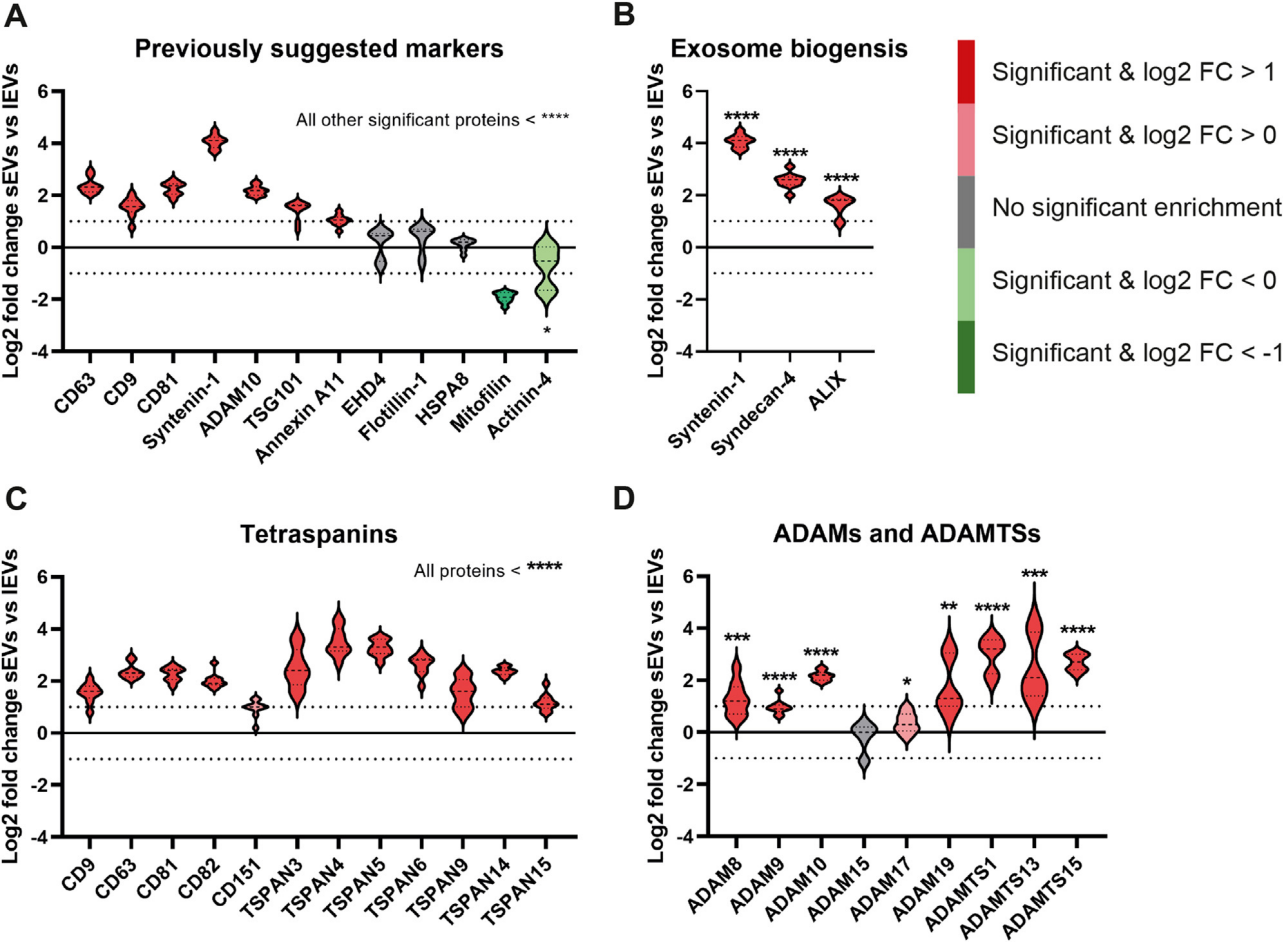
**Tetraspanins and ADAMs/ADAMTSs are enriched in sEVs compared with lEVs.** The log2 fold changes between the sEVs and lEVs determined with quantitative proteomics (tandem mass tag). *A*, twelve proteins that have previously been suggested as markers for sEVs and lEVs (11). *B*, three proteins that have previously been suggested to be part of the biogenesis of the sEV subgroup, exosomes (23). *C* and *D*, all tetraspanins (*C*) and ADAMs/ADAMTSs (*D*) detected in the dataset. *Light red*, significant and fold change >1 (log2 = 0); *dark red*, significant and fold change >2 (log2 = 1) = enriched in sEVs. *Light green*, significant and fold change <−1 (log2 = 0); *dark green*, significant and fold change <−2 (log2 = −1) = enriched in lEVs. *Gray*, no significant enrichment in either sEVs or lEVs. *Dotted lines* on the *y*-axis indicate log2 fold change = 1 and −1 (corresponding to fold change 2 and −2). Data presented as violin plots. N = 9 (N = 3 for each of the three cell lines). lEV, large extracellular vesicle; sEV, small extracellular vesicle.

Recently it was suggested that proteins previously believed to be unique for sEVs were shown not to be enriched in sEVs but equally distributed in all EV subpopulations examined (11). In addition, novel markers for lEVs were suggested by Kowal *et al.* (11). We started by evaluating these suggested proteins (11) in our quantitative proteomic dataset. We validated that CD63, CD9, CD81, Syntenin-1, ADAM10, TSG101, and Annexin A11 were all enriched in sEVs. However, we did not observe enrichment of EHD4 (Fig. 5*A*). In addition, mitofilin and actinin-4 were enriched in lEVs (Fig. 5*A*), validating previous findings (11). Furthermore, we found no significant difference between lEVs and sEVs for flotillin-1 and HSPA8, confirming previous findings from Kowal *et al.* (11) (Fig. 5*A*). Of all these proteins, syntenin-1 had the strongest enrichment with a fold change above 16 in sEVs. This is in line with recent findings showing that syntenin-1 was the most abundant protein in sEVs when 14 cell lines were evaluated (21). Furthermore, Syntenin-1 has previously been found in tetraspanin-enriched microdomains, and to specifically interact with CD63 (22), a protein that was also enriched in sEVs in our quantitative proteomic dataset. We also evaluated the expression of two proteins, syndecan-4 and ALIX, that have been suggested to be involved in the biogenesis of exosomes, together with syntenin-1 (23). These two proteins were also strongly enriched in sEVs, suggesting that our sEV samples at least partly contain exosomes, hence vesicles were released from the multivesicular body (MVB) (Fig. 5*B*).

Tetraspanins were found to be one of the protein groups that was most enriched in sEVs compared with lEVs (Fig. 5*C*). Tetraspanin-3, -4, -5, -6, and -14 were all more enriched in sEVs than the more classical sEV tetraspanins, CD9, CD63, and CD81 (Fig. 5*C*). After validating Kowal *et al.* suggestion that ADAM10 is a novel marker for sEVs, we further investigated the expression of all ADAM (A Disintegrin And Metalloproteases) and ADAMTS (A Disintegrin And Metalloproteinase with ThromboSpondin motifs) proteins quantified in our dataset. We found that, as a group, these proteins were strongly enriched in sEVs compared with lEVs (Fig. 5*D*). While ADAM19 and ADMATS13 were mostly enriched in the D3H1-derived sEVs, ADAMTS1 was mostly enriched in LN and BM-derived sEVs, giving the violin plot for these three proteins a long hourglass shape (Fig. 5*D*). ADAM10, by contrast, had a very similar enrichment in sEVs from all three cell lines, resulting in a tight violin plot (Fig. 5*D*).

**Fig. 6:**
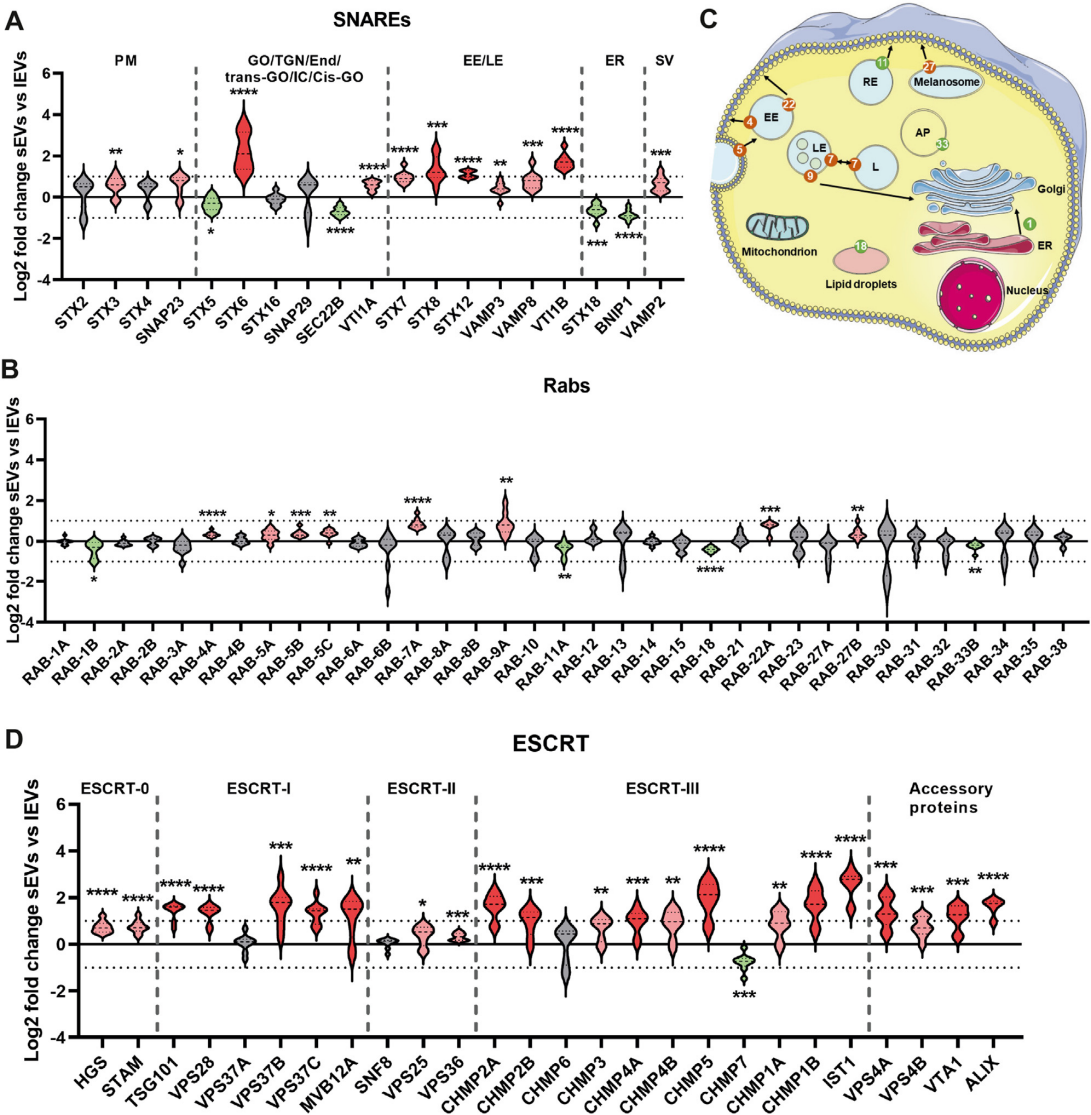
**SNAREs and Rabs associated with endosomes and proteins from the ESCRT machinery are enriched in sEVs compared with lEVs.***A*, the log2 fold change determined with quantitative proteomics of all SNARE proteins detected in the dataset. Location of the SNAREs are according to Hong (57). *B*, the log2 fold change determined with quantitative proteomics of all Rab proteins detected in the dataset. *C*, the location of the enriched Rab proteins according to Hutagalung and Novick (90). Rabs enriched in sEVs and lEVs are labeled with red and green, respectively. *D*, the log2 fold change determined with quantitative proteomics for all proteins that are part of or associated with the ESCRT machinery. *Light red*, significant and fold change >1 (log2 = 0); *dark red*, significant and fold change >2 (log2 = 1) = enriched in sEVs. *Light green*, significant and fold change >−1 (log2 = 0); *dark green*, significant and fold change >−2 (log2 = −1) = enriched in lEVs. *Gray*, no significant enrichment in either sEVs or lEVs. *Dotted lines* on the *y*-axis indicate log2 fold change = 1 and −1 (corresponding to fold change 2 and −2). Data presented as violin plots. N = 9 (N = 3 for each of the three cell lines). AP, autophagosome; *cis*GO, *cis*-Golgi compartments; EE, early endosomes; End, endosomes; ER, endoplasmic reticulum; GO, Golgi apparatus; IC, ER–Golgi intermediate compartments; L, lysosome; LE, late endosomes; lEV, large extracellular vesicle; PM, plasma membrane; RE, recycling endosomes; sEV, small extracellular vesicle; SV, synaptic vesicles; TGN, *trans*-Golgi network; *trans*-GO, *trans*-Golgi compartments.

SNARE (soluble NSF attachment protein receptors) proteins, Rab proteins, and Annexins are large groups of proteins involved in membrane trafficking and vesicle formation such as exocytosis and endocytosis and have previously been suggested to be enriched in sEVs. Analysis of our dataset demonstrated that, although the majority of SNAREs were enriched in sEVs compared with lEVs, there were also SNAREs enriched in lEVs. SNARES involved in membrane fusion in the early and late endosomes were primarily enriched in sEVs, while the SNAREs associated with the ER were enriched in lEVs (Fig. 6*A*). The SNAREs involved with the plasma membrane had a trend of being enriched in sEVs, while some of the Golgi-associated SNAREs were enriched in sEVs, some were enriched in lEVs, and some were equally detected in both EV subpopulations (Fig. 6*A*). The majority of the Rab proteins were not significantly enriched in either subpopulation of EVs. Of the Rab proteins that were significantly enriched in sEVs or lEVs, none demonstrated an enrichment above 2-fold, indicating that these proteins are not suitable to distinguish lEVs and sEVs (Fig. 6*B*). However, detailed analysis showed that Rab proteins involved in the transport of vesicles between the plasma membrane, the early endosome, and the late endosome were enriched in sEVs, while Rab proteins enriched in lEVs were assigned to be located in other organelles (Fig. 6*C*). Of the Annexins that were quantified in this study, only Annexin-A4, -A7, and -A11 were significantly upregulated in sEVs, with only Annexin-A11 demonstrating a change in enrichment above 2-fold (Supplemental Fig. S5*A*).

Heat shock proteins stabilize proteins to ensure correct folding and have also been suggested to be enriched in sEVs. Surprisingly, we found that the majority of the heat shock proteins were not enriched in sEVs but were instead enriched in lEVs (Supplemental Fig. S5*B*).

As a group, integrins were enriched in sEVs; however, they were only enriched in sEVs compared with lEVs in the LN and BM cell lines (Supplemental Fig. S5, *C* and *D*).

The top 20 most enriched proteins in sEVs compared with lEVs were complement components and collagens. This demonstrates that, although the vesicles have been purified on a bottom-loaded density cushion, the most enriched proteins in sEVs compared with lEVs were still most likely contaminating proteins (Supplemental Fig. S5*E*). This finding highlights the coisolation of contaminants with sEVs during high ultracentrifugation isolation and the difficulties to remove them afterward.

Lastly, we analyzed the ESCRT (the Endosomal Sorting Complex Required for Transport) proteins. The ESCRT machinery has been demonstrated to be involved in the release of exosomes, and these proteins have therefore been suggested to be enriched in small EVs. Indeed, we validated that the majority of the ESCRT proteins were enriched in sEVs compared with lEVs in our dataset (Fig. 6*D*).

These findings suggest that proteins involved in the ESCRT machinery and exosome biogenesis, tetraspanins, integrins, ADAMs, and ADAMTSs, are enriched in sEVs compared with lEVs. Furthermore, regarding proteins involved in membrane and vesicle trafficking, such as Rab proteins and SNAREs, primarily those associated with early and late endosomes and their interactions with the plasma membrane were enriched in the sEVs. Heat shock proteins, on the contrary, were enriched in lEVs.

### Large EVs Are Enriched in Ribosomal, Mitochondrial, and Nuclear Proteins as Well as Proteins Involved in Cytokinesis

**Fig. 7:**
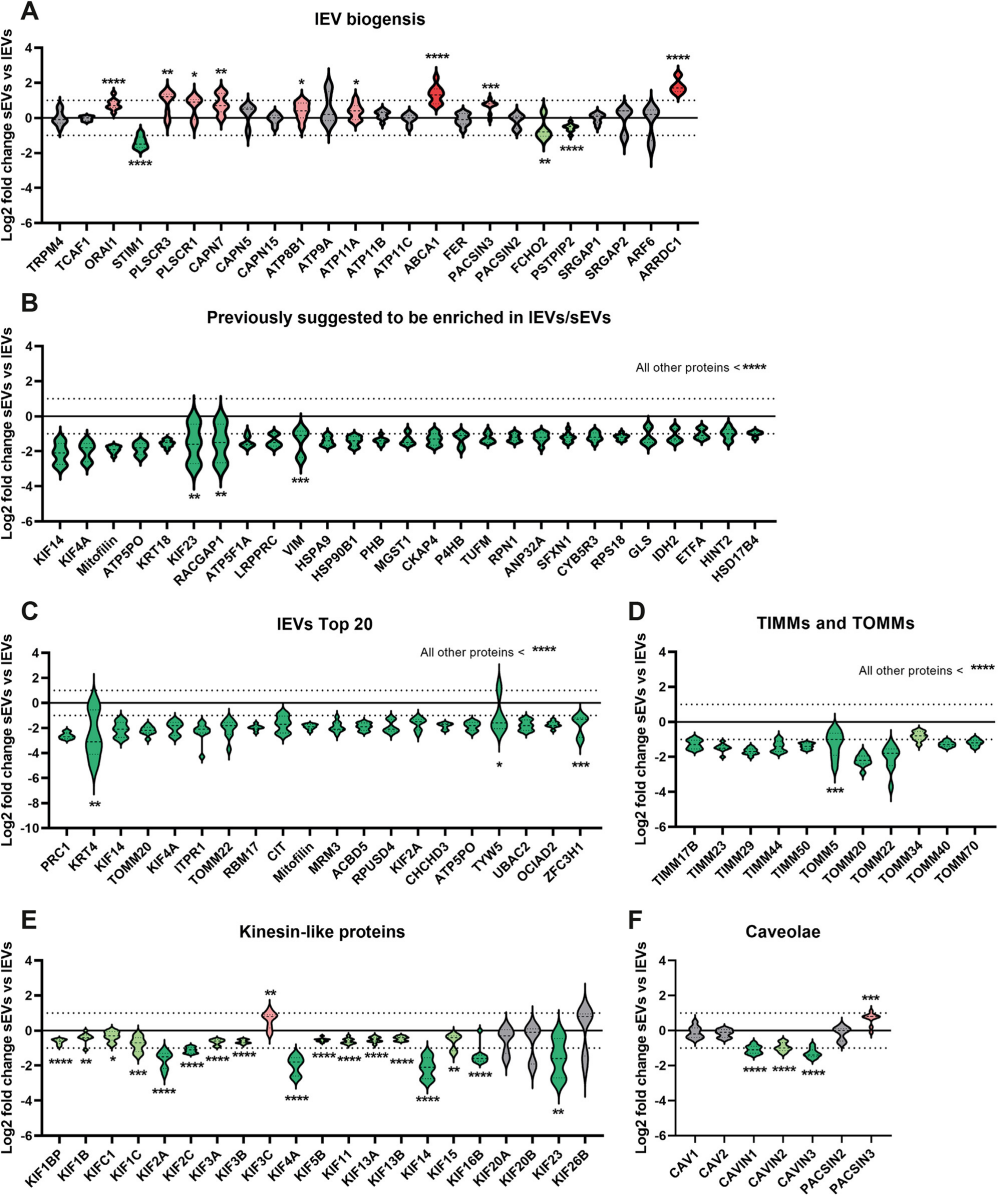
**Mitochondrion- and cytokinesis-associated proteins are enriched in lEVs compared with sEVs.***A*, the log2 fold change determined with quantitative proteomics of proteins that have previously been suggested to be part of the biogenesis of the lEV subgroup, microvesicles/ectosomes (24, 25, 26). *B*, the log2 fold change determined with quantitative proteomics for proteins in our dataset with a log2 fold change below −1 that have previously been suggested to be enriched in the lEV subgroup, microvesicles/ectosomes compared with sEVs (26, 28, 29). *C*, the top 20 most enriched proteins in lEVs based on log2 fold change compared with sEVs. *D* and *E*, the log2 fold change determined with quantitative proteomics for all TIM and TOM proteins (*D*), all Kinesin-like proteins (*E*), and all Caveolae-associated proteins (*F*) quantified in the dataset. *Light red*, significant and fold change >1 (log2 = 0); *dark red*, significant and fold change >2 (log2 = 1) = enriched in sEVs. *Light green*, significant and fold change >−1 (log2 = 0); *dark green*, significant and fold change >−2 (log2 = −1) = enriched in lEVs. *Gray*, no significant enrichment in either sEVs or lEVs. *Dotted lines* on the *y*-axis indicate log2 fold change = 1 and −1 (corresponding to fold change 2 and −2). Data presented as violin plots. N = 9 (N = 3 for each of the three cell lines). lEV, large extracellular vesicle; sEV, small extracellular vesicle.

Less is known about the proteins involved in the biogenesis of lEVs compared with sEVs. We constructed a list of proteins that have been previously suggested to be associated with the release of MVs/ectosomes by budding off of the plasma membrane (24, 25, 26). Briefly, these proteins were associated with Ca^2+^ influx, phospholipid dynamics, and cytoskeletal remodeling. Of these proteins, 24 were quantified in our dataset. Surprisingly, the majority were enriched either in sEVs or there was no significant difference between the two subpopulations (Fig. 7*A*). Only three of these proteins were enriched in our lEVs: STIM1 (Stromal interaction molecule 1), FCHO2 (FCH domain only protein 2), and PSTPIP2 (Proline-serine-threonine phosphatase-interacting protein 2). STIM1 functions as a calcium sensor in the ER, FCHO2 functions in an early step of clathrin-mediated endocytosis, while PSTPIP2 is a member of the Pombe Cdc15 homology (PCH) family of proteins, which coordinates membrane and cytoskeletal dynamics. Several of the violin plots were elongated, which suggests differences between the three cell lines. For example, ARF6, which has been shown to regulate shedding of tumor-derived plasma membrane microvesicles (27), was only significantly enriched in lEVs compared with sEVs from the D3H1 cells. This may suggest that some of these proteins are cell type specific. In addition, we constructed a list of proteins that have previously been shown to be enriched in lEVs compared with sEVs (11, 26, 28, 29). We confirmed a majority of these proteins to be enriched in our lEVs compared with sEVs (Fig. 7*B* and Supplemental Fig. S6*A*). On the other hand, Annexin A1, a protein that has been suggested to be a marker for lEVs shedding from the plasma membrane (30), was only significantly enriched in lEVs compared with sEVs from the D3H1 cells. This may again suggest that some proteins are cell type specific. Next, we analyzed proteins belonging to the same group as some of the proteins that we and others had found to be more enriched in lEVs (Fig. 7*B* and Supplemental Fig. S6*A*), such as septins, alpha-actinins, MICOS complex subunits and ATP synthase subunits, which were all shown to be enriched in lEVs compared with sEVs (Supplemental Fig. S6*B*).

To perform an unbiased analysis, we analyzed the top 20 most enriched proteins in lEVs compared with sEVs and observed that three of the top proteins, PRC1 (Protein regulator of cytokinesis), KIF14 (Kinesin-like proteins KIF14), and KIF4A (Chromosome-associated kinesin KIF4A), can interact with each other and are involved in cytokinesis (31, 32, 33) (Fig. 7*C*). Furthermore, several TOM–TIM complex proteins, involved in translocating proteins into the inner (TIMs) and outer (TOMs) membrane of the mitochondrion, were present in the top 20 list (Fig. 7*C*). We therefore investigated these groups of proteins in more depth. All TIMs and TOMs quantified in the dataset were upregulated in lEVs (Fig. 7*D*). The majority of kinesin-like proteins were also upregulated in lEVs compared with sEVs (Fig. 7*E*).

Furthermore, although none of them demonstrated a 2-fold enrichment, as a group both CCR4-Not (Carbon Catabolite Repression–Negative On TATA-less) and Nuclear Pore Complex proteins were enriched in lEVs compared with sEVs (Supplemental Fig. S7, *A* and *B*). CCR4-Not proteins are involved in gene expression and can be present in both the nucleus and the cytoplasm. Nuclear pore complex proteins are involved in connecting the nucleoplasm and the cytoplasm. In addition, heterogeneous nuclear ribonucleoproteins (hnRNPs) were enriched in lEVs compared with sEVs (Supplemental Fig. S7*C*). These proteins are predominantly present in the nucleus and are involved in controlling the maturation and stability of mRNA (34). Similar to integrins in the sEVs, hnRNPs were mainly upregulated in the lEVs derived from the LN and BM cell lines, and to a lesser extent the D3H1 lEVs (Supplemental Fig. S7*D*).

In addition, both cytosolic (60S and 40S) and mitochondrial (39S and 28S) ribosomal proteins were enriched in lEVs compared with sEVs (Supplemental Fig. S8, *A*–*D*).

Lastly, it was shown that cavins, a group of proteins associated with controlling caveolae formation (Fig. 7*F*), were enriched in lEVs compared with sEVs. However, the other proteins involved in caveolae formation were not enriched in lEVs.

**Fig. 8:**
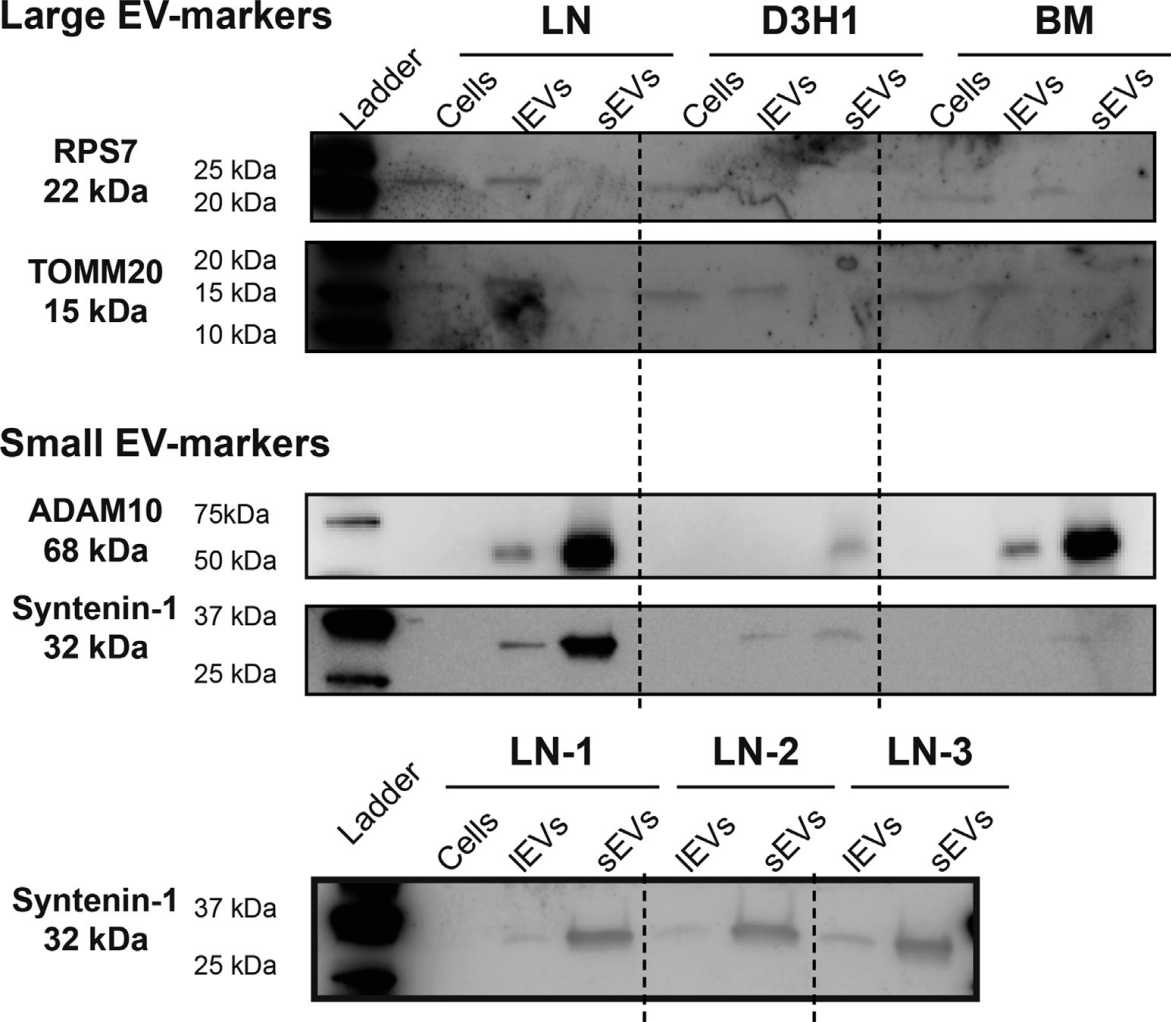
**Validation with Western blot of proteins identified as enriched in large and small EVs with mass spectrometry.** After density cushions equal amount of proteins (5.5 μg) of each sample of the lEVs and sEVs were loaded on SDS-PAGE gels. For cell lysate also 5.5 μg was loaded on the gels. For the only LN sample gel for syntenin-1 10 μg was loaded for all EV samples and 15 μg for the cell lysate. lEV, large extracellular vesicle; sEV, small extracellular vesicle.

These findings demonstrate that proteins associated with the nucleus (nuclear pore complexes, CCR4-Nots, hnRNPs), mitochondria (TIM/TOM complexes, mitochondrial rRNA, MICOS complex subunits, and ATP synthase subunits), ribosomal proteins (60S, 40S, 39S, and 28S), cytokinesis (kinesin-like proteins, RACGAP1, PRC1), heat shock proteins, and cavins were enriched in lEVs compared with sEVs. Western blot was used to validate a few of the proteins identified in the quantitative mass spectrometry analysis. As expected, RPS7 (ribosomal proteins) and TOMM20 (TIMM/TOMMs) were enriched in the lEVs and syntenin-1 (exosome biogenesis) and ADAM10 (ADAMs/ADAMTSs) were enriched sEVs, validating the mass spectrometry analysis (Fig. 8). In addition, the Western blots in Figure 2 for CD63, CD81, CD9, and flotillin-1 validate the mass spectrometry results by showing that all three tetraspanins and ADAM10 are enriched in sEVs, while no difference can be observed for flotillin-1 between the two EV subpopulations.

**Fig. 9:**
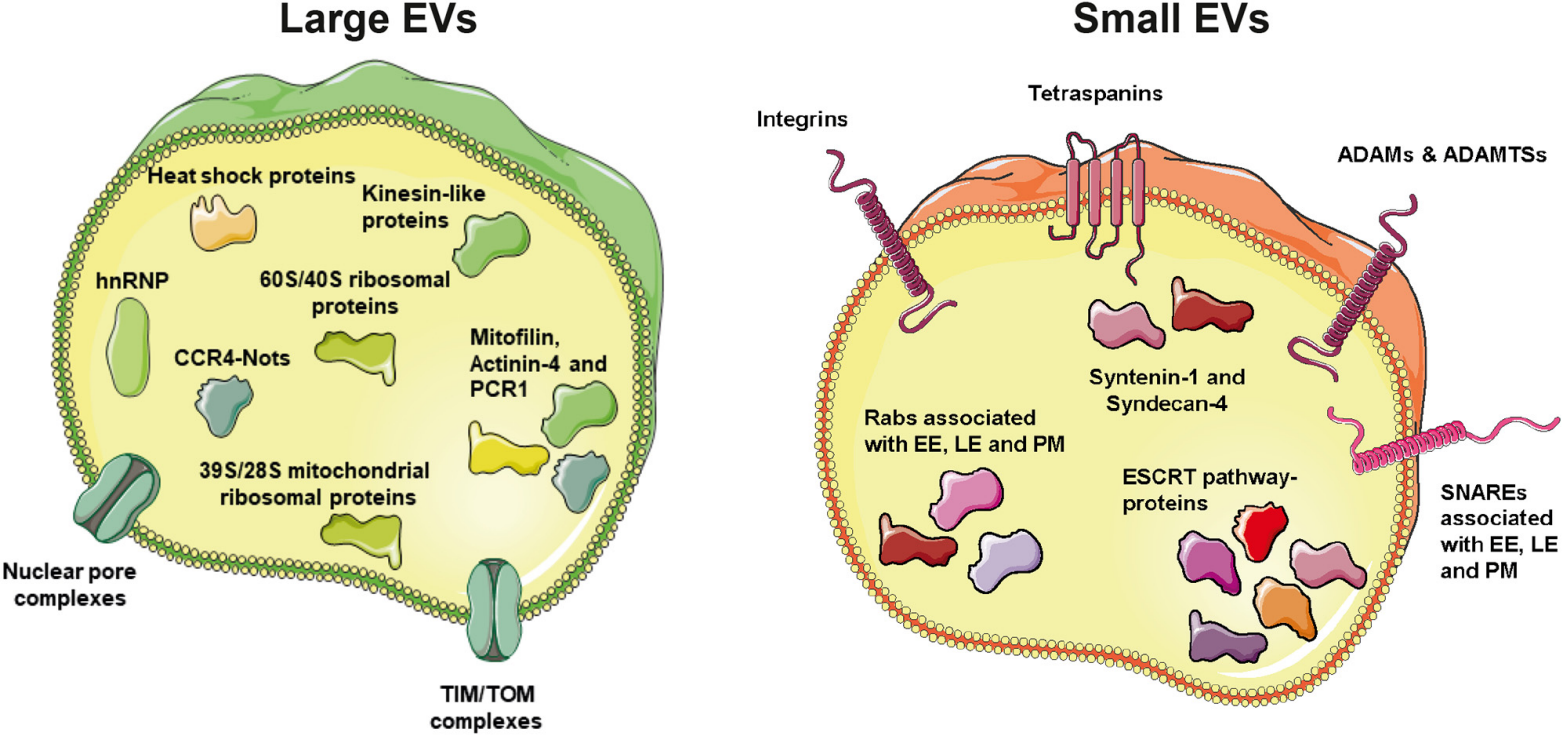
**Proteins enriched in large and small EVs.** Schematic illustration showing the proteins enriched in large EVs and small EVs as determined by quantitative proteomics. EE, early endosomes; EV, extracellular vesicle; LE, late endosomes; PM, plasma membrane.

The protein groups shown to be enriched in sEVs and lEVs, respectively, are summarized in Figure 9.

### The Protein Cargo of Small and Large EVs Is Different in High Compared With Low Metastatic Cell Lines

**Fig. 10:**
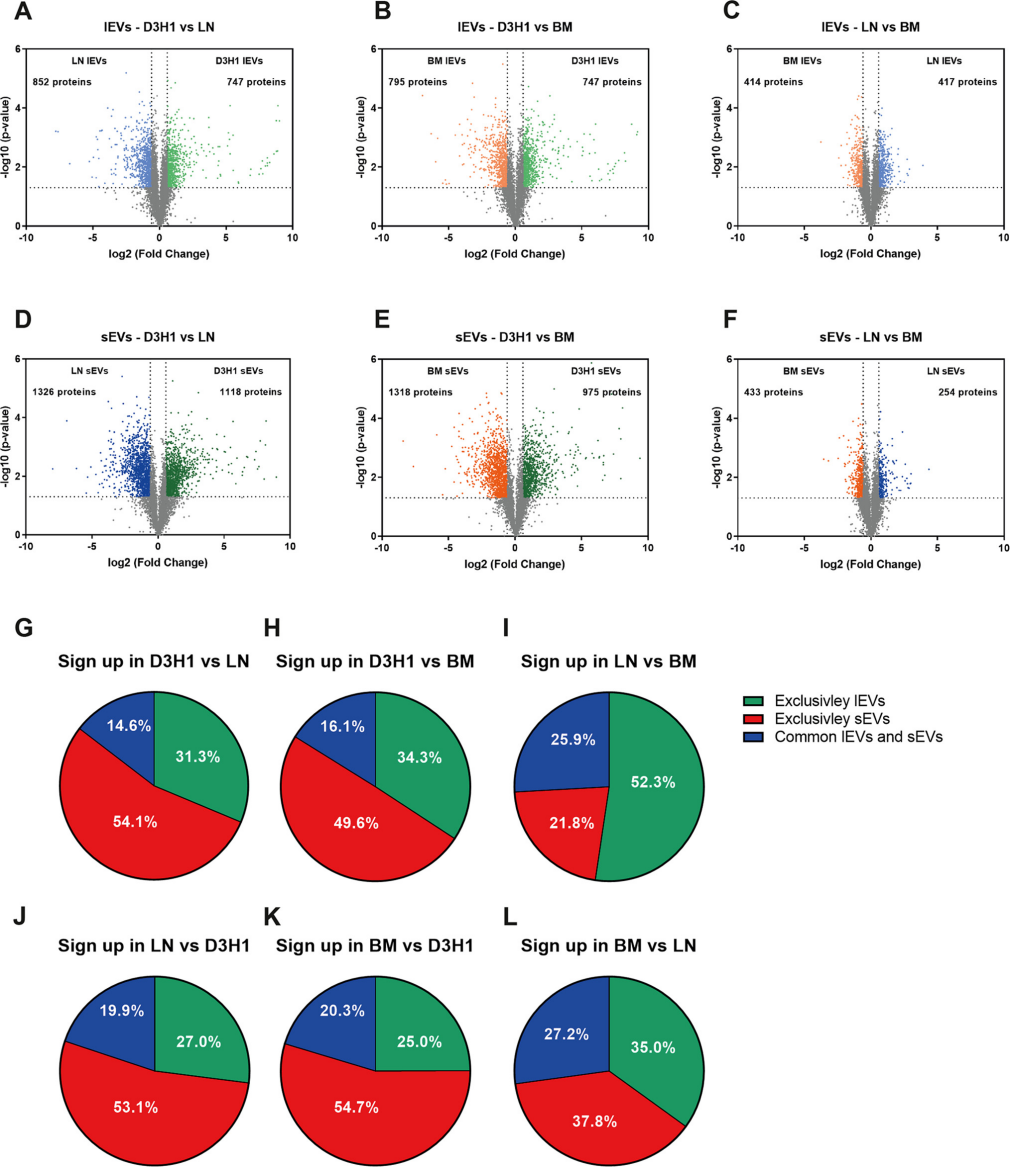
**Alteration in the protein cargo of small and large EVs released by low and high metastatic cell lines.** The quantitative proteomic (tandem mass tag) dataset was also used to determine the differences in the proteomes of lEVs and sEVs between the three cell lines. Three biological replicates (45 μg protein/sample) were used from three different cell lines, resulting in N = 3. *A–F*, volcano plots of the proteomes of lEVs in LN *versus* D3H1 (*A*), BM *versus* D3H1 (*B*), LN *versus* BM (*C*) and sEVs in LN *versus* D3H1 (*D*), BM *versus* D3H1 (*E*), and LN *versus* BM (*F*) identified proteins significantly enriched more than 1.5 fold change. *Dotted lines* indicate cutoffs: 1.3 on the *y*-axis (corresponding to *p* < 0.05) and 0.585 on the *x*-axis (corresponding to fold change > 1.5). *G–L*, the significantly altered proteins in lEVs and sEVs for each cell line were compared to determine if the altered proteins were exclusively altered in lEVs, exclusively altered in sEVs, or altered in both lEVs and sEVs as cells became more metastatic. lEV, large extracellular vesicle; sEV, small extracellular vesicle.

The three breast cancer cell lines used in this study have different metastatic phenotypes. D3H1 is a low metastatic cell line, while LN and BM are high metastatic cell lines isolated from a lymph node and brain metastasis, respectively (18, 19). We compared the EVs from the different cell lines to determine if the same proteins were altered in both sEVs and lEVs when breast cancer cells become more metastatic, although this was not the main aim of this study. We found that EVs from the two high metastatic cell lines were most alike and significantly different from the EVs derived from the low metastatic cell line, both for the sEVs and lEVs (Fig. 10, *A–F*). Furthermore, the overlap of proteins enriched in both sEVs and lEVs for each comparison was low (14.6–27.2%), suggesting that different proteins are loaded into lEVs and sEVs when breast cancer cells become more metastatic (Fig. 10, *G–L*).

## Discussion

Robust protein markers for EV subpopulations are lacking, restricting the possibility to dissect the biological functions, therapeutic effects, and biomarkers for specific EV subpopulations in many current EV studies. We present here an in-depth analysis of the proteomes of lEVs and sEVs to address this knowledge gap. We first characterized the lEVs and sEVs and found that the buoyant density of both subpopulations was similar (∼1.1 g/ml). Quantitative proteomics showed that the proteins enriched in lEVs compared with sEVs were associated with the mitochondrion (TIM/TOM complexes, mitochondrial rRNA, and mitofilin), nucleus (nuclear pore complexes, CCR4-Nots, and hnRNPs), cytokinesis (kinesin-like proteins), ribosomes (60S, 40S, 39S, and 28S), and heat shock proteins. The proteome of sEVs was enriched in proteins involved in the ESCRT machinery, proteins involved in exosome biogenesis, tetraspanins, integrins, and ADAMs, as well as Rab proteins and SNAREs, associated with early and late endosomes and their interactions with the plasma membrane (Fig. 9). We validated several proteins previously suggested to be enriched in either smaller or larger EVs (11, 26, 28, 29) and suggest several new proteins that could be used as markers to distinguish between these two subpopulations in the future.

Both the tetraspanins and ADAMs/ADAMTSs protein groups were heavily enriched in sEVs compared with lEVs. It is well known that tetraspanins such as CD9, CD63, and CD81 are enriched in microdomains, in the intraluminal vesicles of the multivesicular endosomes, and in sEVs (11, 35, 36). However, less is known about other tetraspanins and EVs, although Tetraspanin-9 and -14 have previously been identified exclusively in sEVs compared with lEVs (26). Furthermore, Tetraspanin-8 has been suggested to be implicated in exosome uptake (37), while Tetraspanin-6 has been shown to regulate sEVs release (38, 39). In addition to CD9, CD63, and CD81, we found that tetraspanin-3, -4, -5, -6, -9, -14, and -15 were enriched in sEVs, many of them to a greater extent than CD9. Although different cell types have distinct TspanC8 (Tetraspanin-5, -10, -14, -15, -17, and -33) repertoires (40), our analysis suggests that, in addition to CD63, CD9, and CD81, several other tetraspanins, such as tetraspanin-4, -5, -6, and -14, can also work as markers enriched in sEVs.

ADAMs are transmembrane proteases that cleave off the ectodomain of membrane proteins. This shedding mechanism is essential for cytokine secretion, cell–cell adhesion, and signaling by transmembrane ligands and receptors (41). Although they are also proteases, ADAMTSs are secreted extracellular enzymes, in contrast to the membrane-bound ADAMs. Known functions of ADAMTS proteases include tissue development and maintenance and processing of procollagens, von Willebrand factor, and other extracellular matrix proteins (42). ADAMs and ADAMTSs have been found in EVs derived from several different cell types (43), and ADAM10 has been shown to be enriched in sEVs compared with lEVs (11, 26, 29). Of the nine ADAMs and ADAMTSs that we quantified in our dataset, eight were enriched in sEVs compared with lEVs, suggesting that ADAMs and ADAMTSs are enriched as groups in sEVs. It has also been shown that ADAM10 is associated with tetraspanins such as CD81 and CD9 (44, 45), which were also enriched in sEVs in our dataset. Furthermore, members of the TspanC8 subfamily have been shown to positively regulate ADAM10 surface expression levels and affect ADAM10-dependent Notch activation and the cleavage of several ADAM10 substrates (46). In addition, CD9 has been shown to regulate the sheddase activity of ADAM17 (47). Together, this suggests a close relationship between ADAMs and tetraspanins that may be of interest for future EV studies to determine in more detail.

Rab GTPases are a large family of proteins that control intracellular membrane traffic by regulating vesicular transport as well as docking and fusion to the target organelle membrane. We found that RAB-1B, -11A, -18, and -33B were significantly enriched in lEVs compared with sEVs. RAB-18 facilitates membrane traffic between ER and lipid droplets (48) and has previously been shown to be enriched in lEVs compared with sEVs (26). RAB-11A is involved in the transport of recycling endosomes toward the plasma membrane, but its role in the release of sEVs has been debated; some studies have demonstrated a role, while others did not see an effect on sEV-release when shRNA for RAB-11A was used (49, 50, 51). However, the role of RAB-11A in the biogenesis of lEVs is still unknown. In our study, RAB-4A, -5A, -5B, -5C, -7A, -9A, -22A, and -27B were significantly enriched in sEVs compared with lEVs. Several of these Rabs have been shown to affect secretion of sEVs (51, 52) and, to a lesser extent, lEVs (53). RAB-7A and RAB-27B have also been shown to be essential for vesicle-mediated secretion of miRNAs from endothelial cells (54). It is important to note that proteins not enriched in an EV subpopulation can still participate in their biogenesis, and the presence of a protein does not necessarily mean it has been part of their biogenesis. However, importantly, no significant difference between lEVs and sEVs was observed for the majority of Rab proteins and the fold changes that were significant were very small. This suggests that Rab proteins are not good markers to distinguish these two EV subpopulations and may be important for the biogenesis of both EV subpopulations.

SNARE proteins are associated with different organelles where they mediate membrane fusion at these compartments. Of interest, all the SNAREs associated with the early and late endosomes were enriched in our sEVs. In addition, we found that SNAP23 was enriched in our sEVs. SNAP23 is mainly associated with the plasma membrane but has previously been suggested to be involved in the fusion of MVBs with the plasma membrane and hence participate in the release of exosomes (55). SNAP23 has been shown to colocalize with STX6, which was one of the most enriched SNAREs in our sEVs (56). STX5, SEC22B, STX18, and BNIP1 have mainly been associated with the organization of ER subdomains and the transport between the ER and Golgi (57, 58, 59), and these were the only four SNAREs enriched in lEVs. Furthermore, RAB-18 has been shown to be associated with STX18 and BNIP1 to form an ER to lipid droplet contact (60) and was one of few Rab proteins we found to be enriched in lEVs compared with sEVs. These findings may suggest a link between lEVs and ER, which should be further investigated in future studies.

ESCRT consists of four complexes (ESCRT-0, -I, -II, -III) and accessory proteins. These proteins promote and facilitate membrane budding away from the cytoplasm. For example, the ESCRT machinery is involved in the cleavage of membranes shared by two daughter cells during cell division, viral budding, and the budding of intraluminal vesicles into endosomes leading to the formation of MVBs. For this reason, the ESCRT machinery has long been linked to the biogenesis of exosomes. The ESCRT-I protein, TSG101, has also been shown to be recruited by ARRDC1 to the plasma membrane where it promotes the release of microvesicles (61). Other ESCRT proteins have been shown to take part in vesicle budding directly from the plasma membrane of T cells (62). Hence, ESCRT proteins have also been suggested to play a role in the release of EVs directly from the plasma membrane. However, in our dataset all but four of the ESCRT machinery proteins were enriched in sEVs compared with lEVs, with only one protein found to be enriched in lEVs.

It was evident that crude sEVs were more contaminated by soluble proteins than crude lEVs. This was expected, as higher centrifugation speed and longer centrifugation time is needed to pellet the sEVs, which allows more soluble proteins to coisolate with the vesicles. We have also previously observed this for tissue EVs, as the crude lEVs clustered with the density gradient purified lEVs, while the crude sEVs did not cluster with the density gradient purified sEVs, suggesting that the proteome was more altered/purified in sEVs than lEVs after a density gradient purification step (13). Taken together, these findings highlight the need to purify crude sEVs in particular prior to proteomic analysis.

While the proteins enriched in sEVs were strongly associated with the plasma membrane, the early endosome, and late endosome, the proteome of lEVs were associated with other organelles such as the mitochondrion and nucleus. For example, mitochondrial proteins such as TIM/TOM proteins, mitochondrial rRNA, ATP synthase, and mitofilin were enriched in lEVs. Mitofilin has previously been shown to be enriched in lEVs (11) and is located in the inner membrane of the mitochondrion where it is part of the MICOS complex, which is crucial for maintaining the crista junctions (63). Interesting, another MICOS complex protein, CHCHD3, was also enriched in our lEVs. ATP5F1A is another protein that has previously been shown to be enriched in lEVs compared with sEVs (28, 29). ATP5F1A is part of a large enzyme complex called ATP synthase, which is located in the mitochondrial inner membrane, where it catalyzes the formation of APT. Similarly to the MICOS complex, we identified several ATP synthases enriched in lEVs compared with sEVs. We and others have previously identified mitochondrial DNA (16, 64, 65), RNA (14), and proteins (29, 66) in different subpopulations of EVs. We suggest here that, although mitochondrial protein, RNA, and DNA might be present in several subpopulations of EVs, mitochondrial proteins are enriched in lEVs, which supports previous findings (26, 29). It has previously been shown that endosomes directly interact with mitochondria in erythrocytes (67), blood contains cell-free intact circulating mitochondrion (68), lEVs can contain intact mitochondrion (69), and mitochondria can generate vesicles that transport proteins to the lysosome and peroxisomes (70, 71, 72). Together, these findings may suggest that there is a connection, interaction, or overlap between processes in the mitochondrion and the biogenesis of certain subpopulations of lEVs.

We found that the nucleus was linked to the proteins enriched in lEVs compared with sEVs, through protein groups such as CCR4-Not proteins, nuclear pore complex, and hnRNP. CCR4-Not complex is a multisubunit protein complex involved in regulating gene expression by regulating RNA metabolism from the synthesis of RNA in the nucleus to the decay of RNA in the cytoplasm. Although CCR4-Not proteins have been previously detected in EVs (Vesiclepedia.org 30th of November 2020), little is known of their function in this capacity.

hnRNPs are a large family of functionally diverse RNA bindings proteins. They are involved in several RNA-associated processes such as pre-mRNA processing, splicing, and transport from nucleus to the cytoplasm. Several members of this family have been suggested to play a role in the loading of RNA into sEVs. For example, HNRNPA2B1 has been shown to specifically bind to miRNAs and long noncoding RNA and to control the loading of these RNAs into sEVs (73, 74, 75). Furthermore, HNRNPA1 (76, 77), HNRNPC1 (78), and SYNCRIP (79) have been suggested to load miRNA into sEVs. Only one study has suggested a role for hnRNPU in sorting miRNA into lEVs (80). While the majority of previous studies on hnRNPs and EVs have focused on sEVs, we found that hnRNP was enriched in lEVs compared with sEVs.

Furthermore, hnRNPs and integrins were two protein groups that behaved differently in the low metastatic cell line (D3H1) compared with the two high metastatic cell lines (LN and BM). Both these groups of proteins have been suggested to be associated with cancers (81, 82). It has also been shown that the integrins of tumor sEVs determine their organotropic metastasis (83). Furthermore, the two highly metastatic cell lines released relatively more sEVs than lEVs, while the low metastatic cell line released relatively more lEVs than sEVs, both when protein and particles were measured. We also observed that the majority of alteration in protein cargo was seen for the sEVs when we compared the low metastatic and high metastatic cell-derived EVs. Although our dataset with only one low metastatic and two high metastatic cell line is too small to draw large conclusions, these alterations warrant further studies into how different EV subpopulations are altered in concentration and protein cargo when tumor cells become more metastatic and highlight the importance of analyzing different subpopulations separately and not as a bulk or a mix.

Cytokinesis is the last stage of the cell division process and is where the cytoplasm splits into equal halves and the cell becomes two daughter cells (33, 84). Several proteins take part in orchestrating this important event. Several of the top enriched proteins in the lEVs, including KIF4A, KIF14, PRC1, RACGAP1, and KIF23, have been shown to take part in this event (33, 84). For example, RACGAP1 has been shown to interact with KIF23 to form the centralspindlin complex, which is essential for the formation of the central spindle. RACGAP1 also interacts with PRC1 to stabilize and maintain the central spindle as anaphase proceeds (84). A recent paper showed that KIF23 and RACGAP1, as part of the midbody remnant formed during cytokinesis, were part of a subpopulation of EVs in colon cancer that could promote an invasive phenotype in fibroblasts (85). Although several studies, including our current study, suggest that these proteins are enriched in lEVs compared with sEVs (28, 29, 86), Rai and colleagues suggest that this class of EVs is distinct from both sEVs and lEVs (85). This certainly warrants further investigations into these proteins and their potential role in the biogenesis and potential usage as markers for one or several subpopulations of EVs.

In conclusion, our study shows that the proteome of large and small EVs is substantially dissimilar, although their densities are similar. Tetraspanins, ADAMs, ADAMTSs, and ESCRT proteins, as well as SNAREs and Rab proteins associated with endosomes, were enriched in sEVs compared with lEVs. On the other hand, ribosomal, mitochondrial, and nuclear proteins as well as proteins involved in cytokinesis were enriched in lEVs compared with sEVs. However, proteins such as Flotillin-1, the majority of the Rab proteins, and annexins were not differently expressed in the EV subtypes. However, we cannot exclude that additional subpopulations exist within our two EV subpopulations, and future studies will have to dissect this further. Here, we lay a foundation of suggested EV markers for future investigations. This is an important piece of information for the EV field as better markers are needed to evaluate which EV subpopulations have been isolated and analyzed to fully understand the EV secretome and its functions and interactions.

## Data Availability

The MS proteomics data have been deposited to the ProteomeXchange Consortium *via* the PRIDE partner repository with the dataset identifier PXD029212 (87, 88). We have submitted all relevant data from our experiments to the EV-TRACK knowledgebase (EV-TRACK ID:EV210213) (89).

## Supplemental data

This article contains supplemental data (26, 28, 29).

## Conflict of interest

C. L. owns equity in Exocure Bioscience Inc.

## Supplemental Data

Supplemental Figures S1–S8

Supplemental Figures and Table Captions
