# Supplementary material for: Quantitative Proteomics Identifies Proteins Enriched in Large and Small Extracellular Vesicles
Source: Mol Cell Proteomics. 2022 Jul 30;21(9):100273. doi: 10.1016/j.mcpro.2022.100273 (PMC9486130; doi:10.1016/j.mcpro.2022.100273)

Supplementary Figure 1

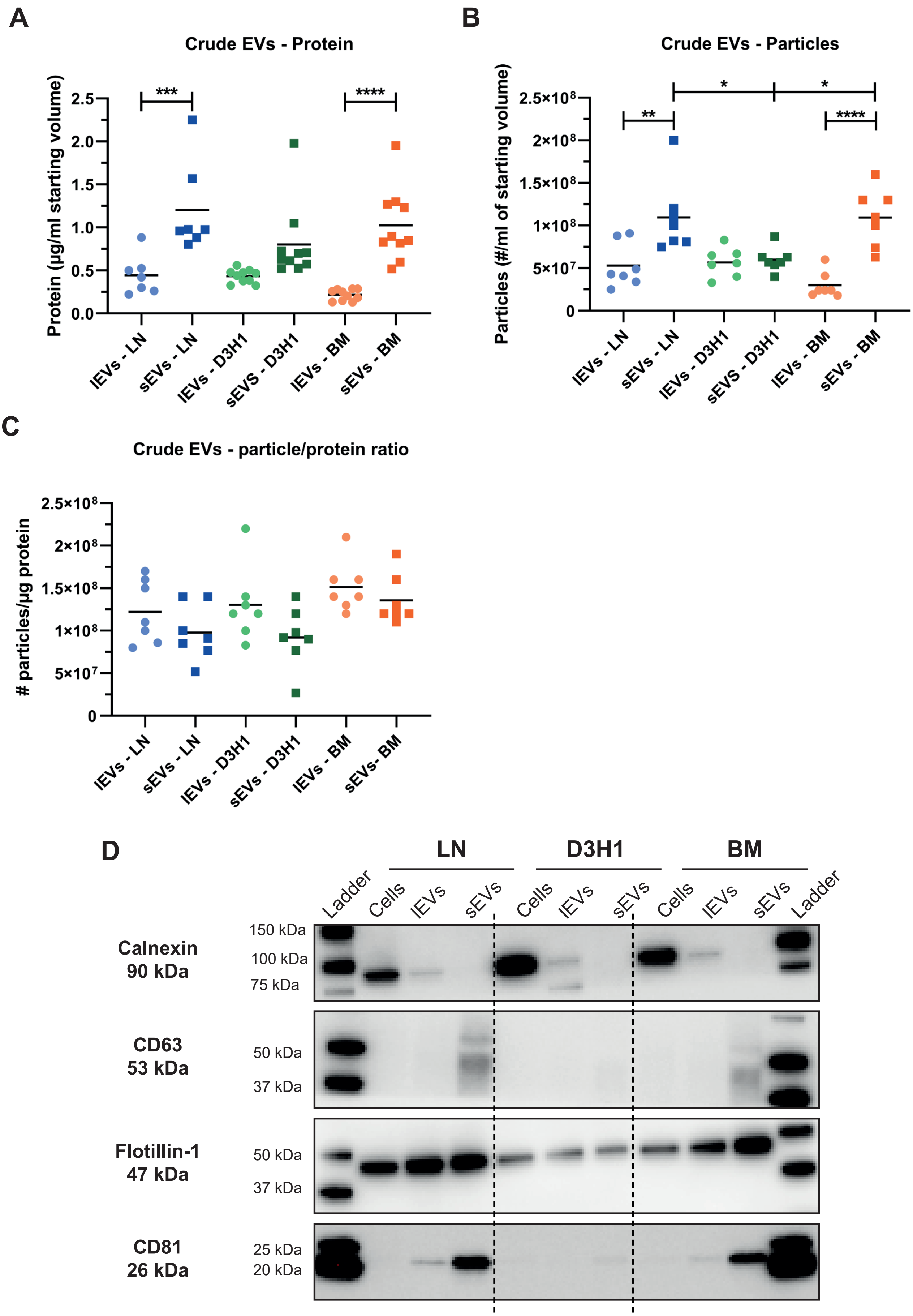

Supplementary Figure 2

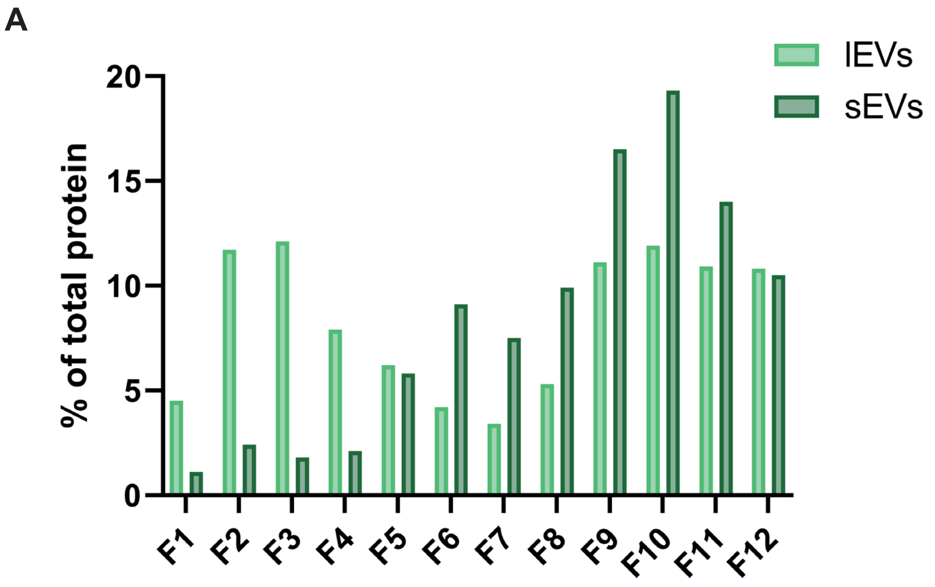

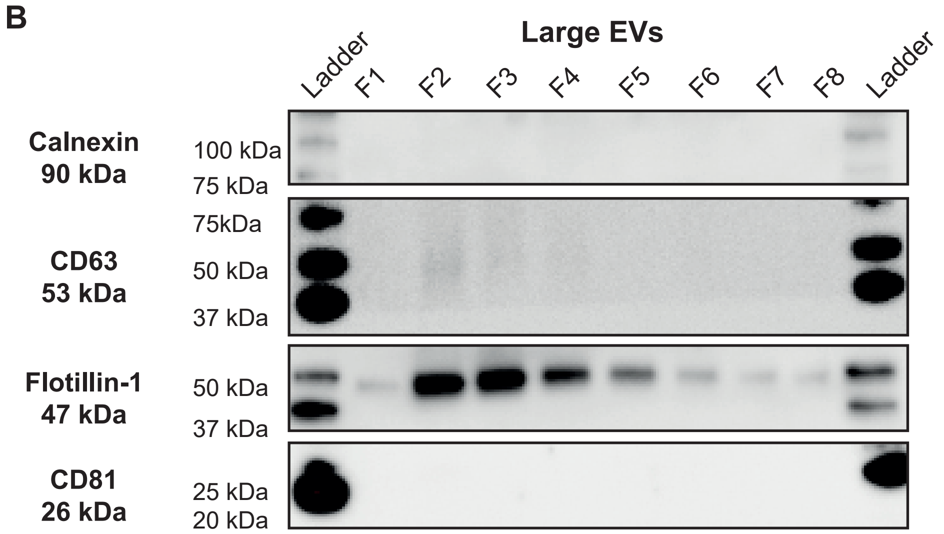

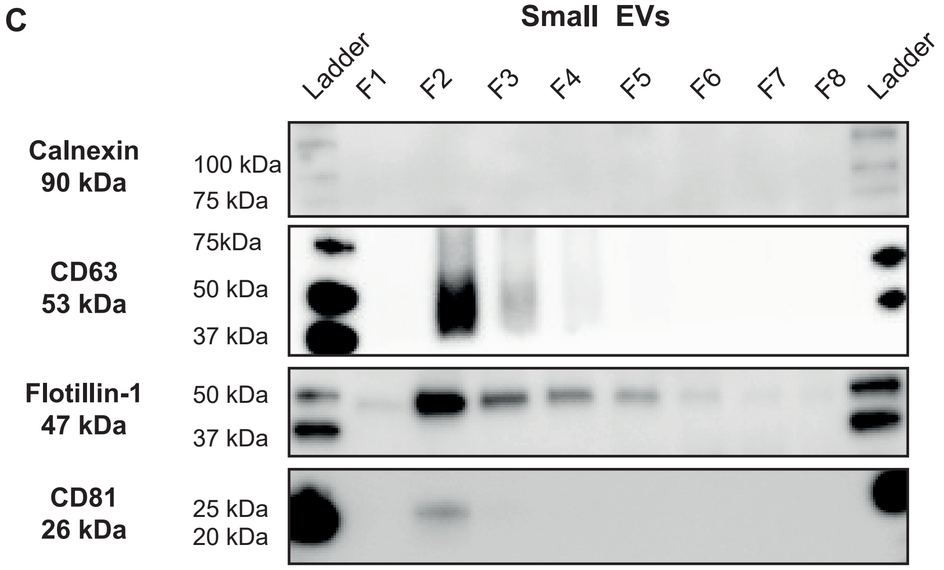

Supplementary Figure 3

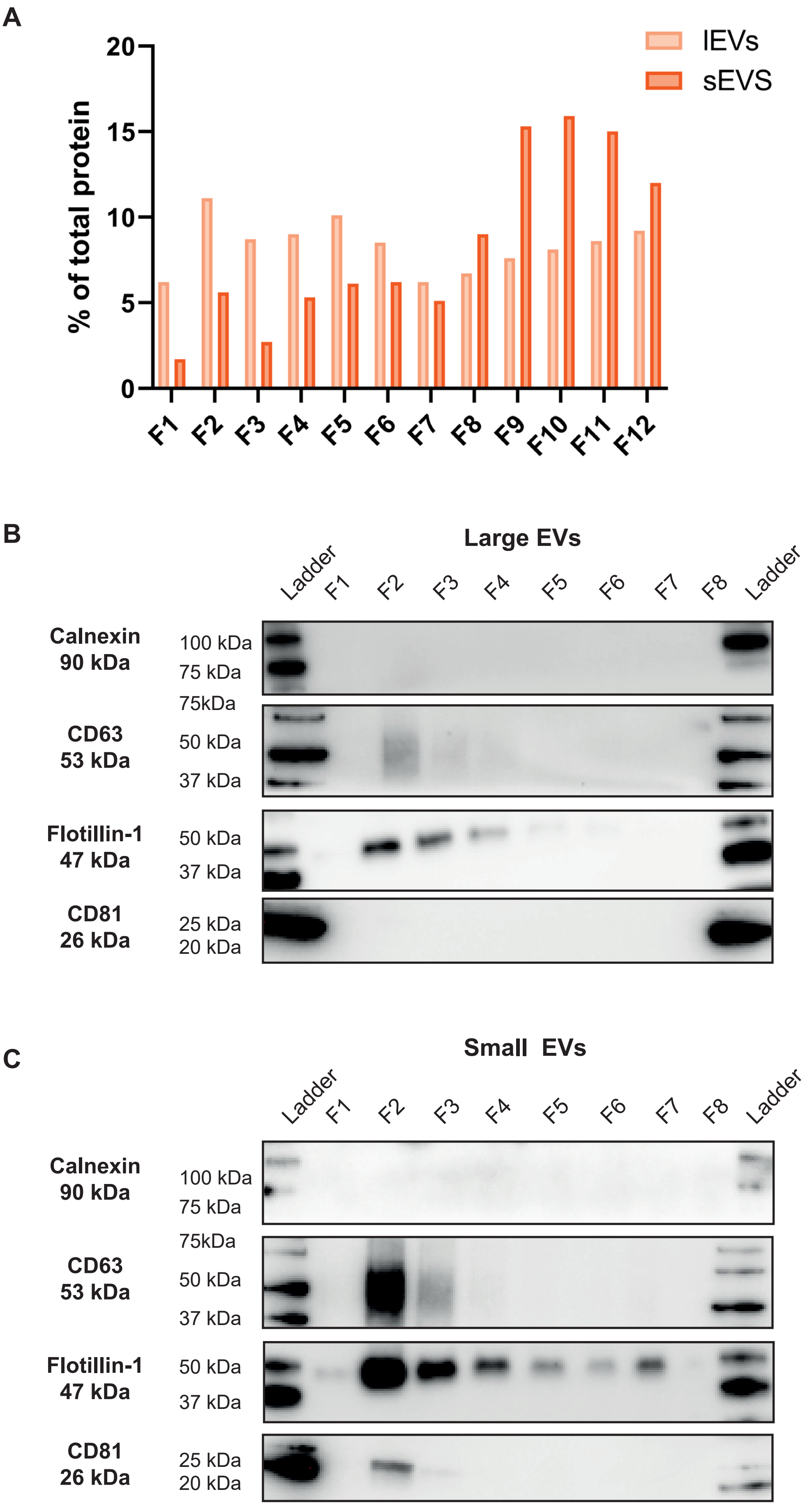

Supplementary Figure 4

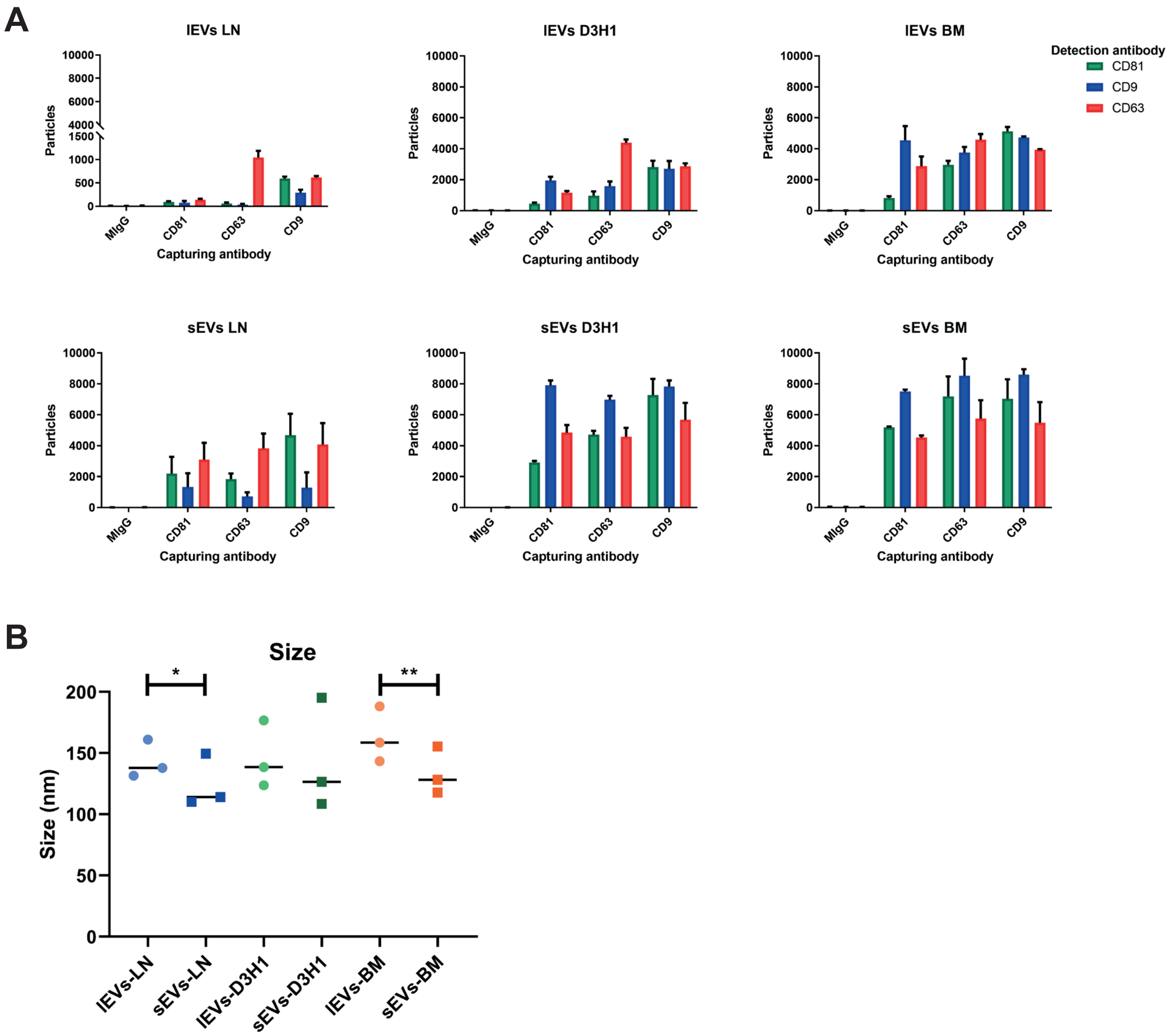

Supplementary Figure 5

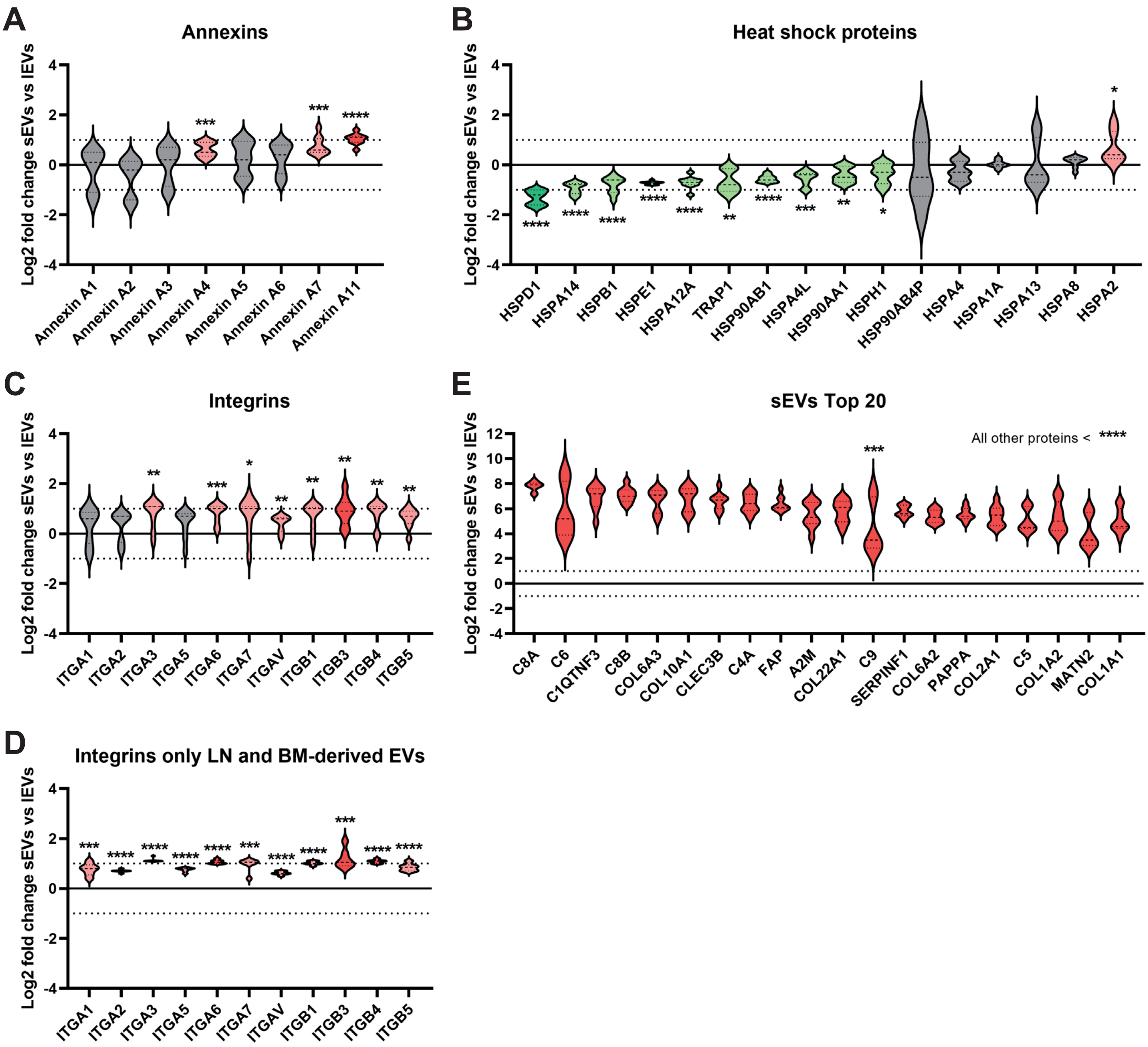

Supplementary Figure 6

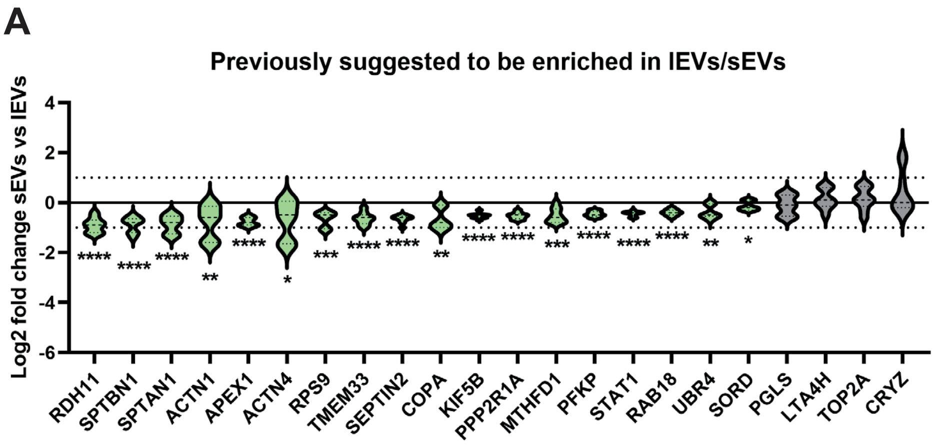

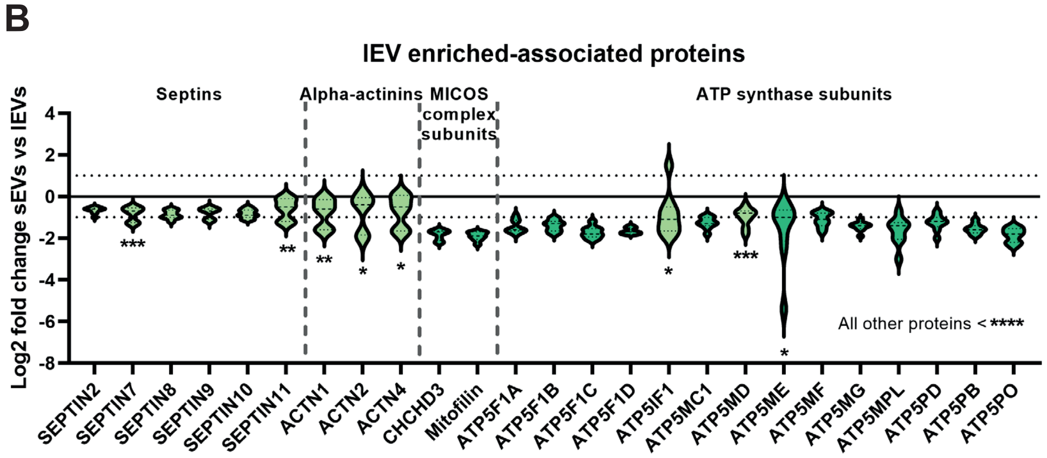

Supplementary Figure 7

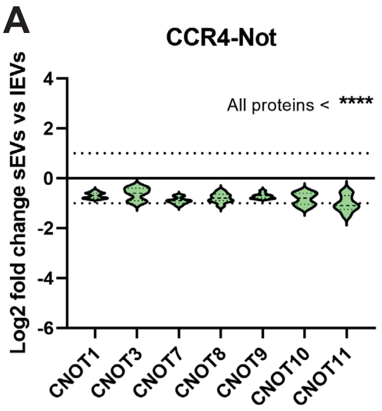

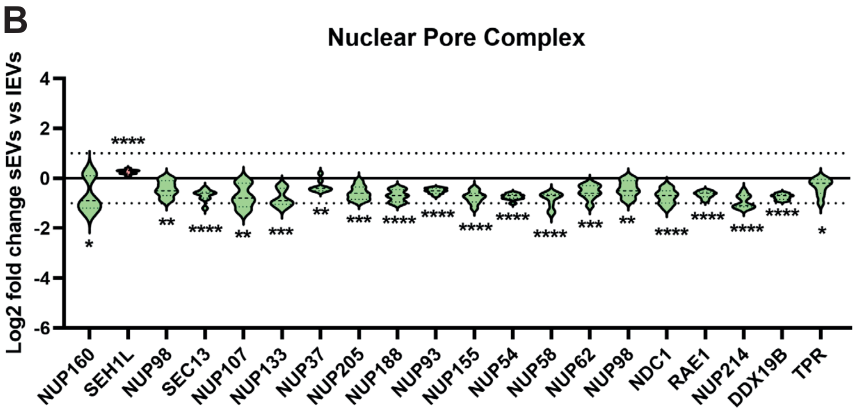

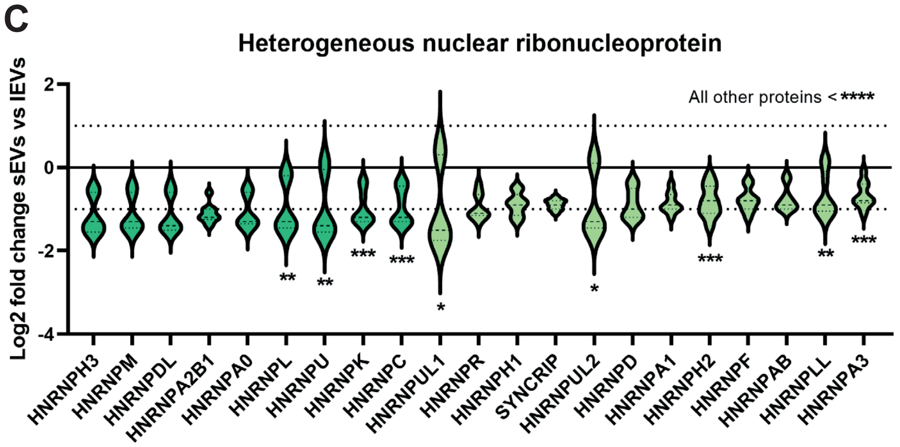

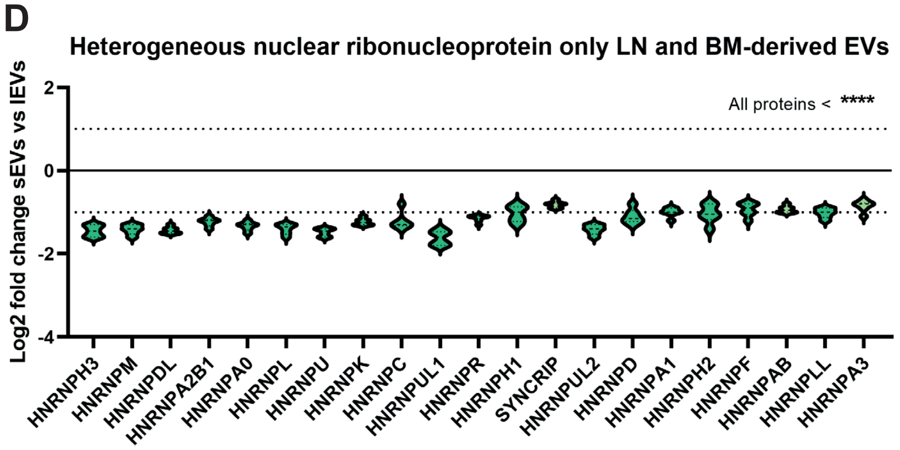

Supplementary Figure 8

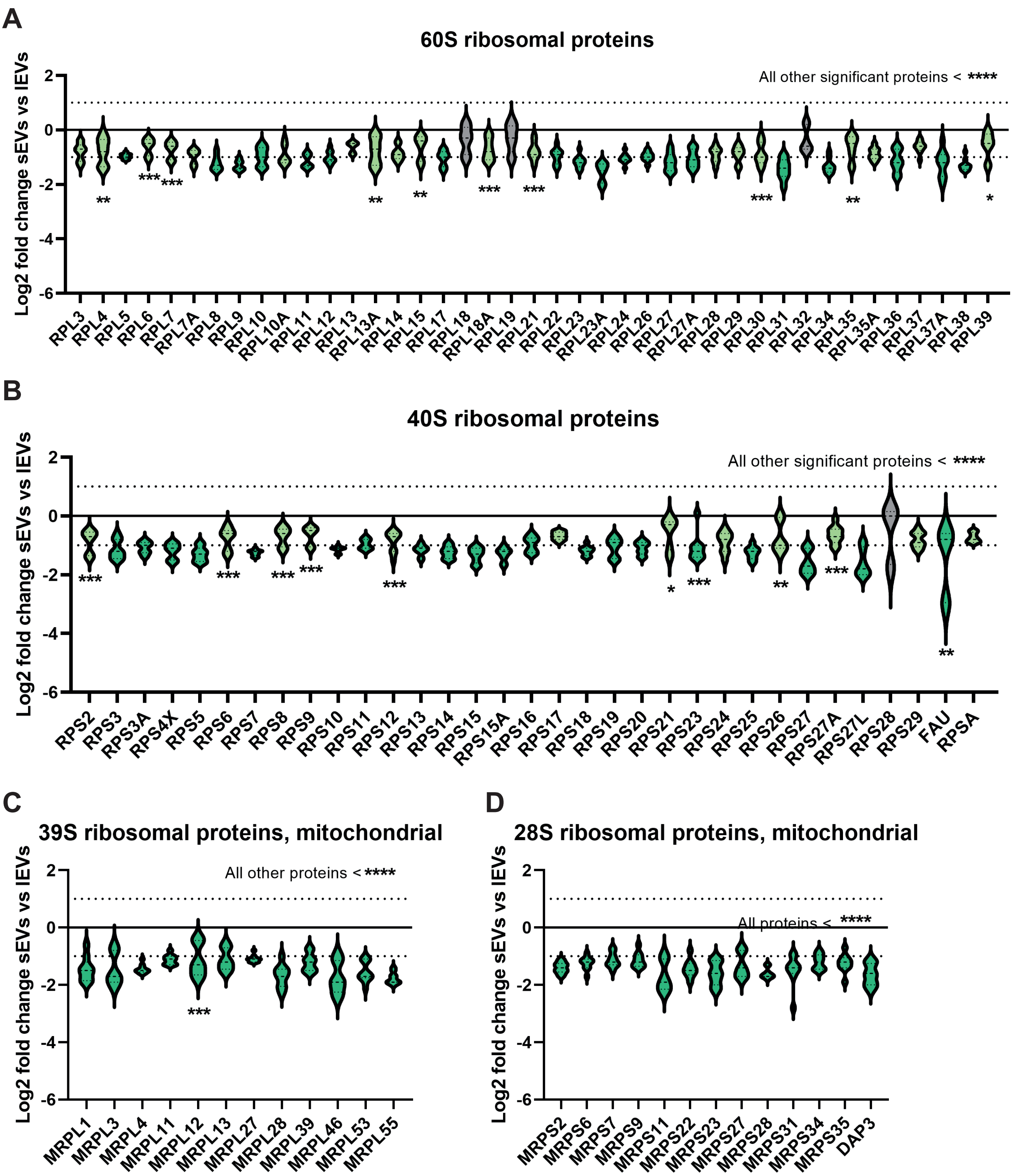

Supplement: Supplemental Figures S1–S8 [file mmc2.pdf]
