# Supplementary material for: Quantitative Proteomics Identifies Proteins Enriched in Large and Small Extracellular Vesicles
Source: Mol Cell Proteomics. 2022 Jul 30;21(9):100273. doi: 10.1016/j.mcpro.2022.100273 (PMC9486130; doi:10.1016/j.mcpro.2022.100273)
Supplement: Supplemental Figures and Table Captions [file mmc3.docx]

Supplementary Figure 1. Evaluation of the crude lEVs and sEVs. (A-B) Amount of proteins (A) and particles (B) in the crude EV-enriched pellets after 16,500 × g (lEVs) and 118,00 × g (sEVs) centrifugation per ml starting cell culture media from all three cell lines. N = 7-10, ordinary one-way ANOVA and Sidak’s multiple comparison test. * p-values = 0.05, *** p-values = 0.001, **** p-values = 0.0001. (C) Particle to protein ratio for all crude EV-enriched samples for lEVs and sEVs from all three cell lines. N=7-10, ordinary one-way ANOVA and Sidak’s multiple comparison test. (D). Western blot was used to investigate the presence of the classical vesicle markers flotillin-1, CD63, CD9 and CD81, as well as the endoplasmic reticulum protein, calnexin. A total of 4.8 µl (1.5-10.2µg protein) was loaded per sample.

Supplementary Figure 2. Evaluation of the density gradient for lEVs and sEVs derived from D3H1 cells. (A) Concentration of proteins in the D3H1 iodixanol gradient fractions determined with Qubit. Data presented as the percentage of the total amount of particles or proteins in fractions 1–12. N=1. (B-C) After density flotation and fractionation of D3H1 lEVs and sEVs in high-resolution iodixanol gradients, equal volumes (36 µL) of each fraction (F1–F8) were loaded onto SDS-PAGE gels.

Supplementary Figure 3. Evaluation of the density gradient for lEVs and sEVs derived from BM cells. (A) Concentration of proteins in the BM iodixanol gradient fractions determined with Qubit. Data presented as the percentage of the total amount of particles or proteins in fractions 1–12. N=1. (B-C) After density flotation and fractionation of D3H1 lEVs and sEVs in high-resolution iodixanol gradients, equal volumes (36 µL) of each fraction (F1–F8) were loaded on SDS-PAGE gels.

Supplementary Figure 4. Analysis of the presence of tetraspanins and size of lEVs and sEVs by ExoViewTM and NTA. A) ExoViewTM showing the presence of CD81, CD63 and CD9 on lEVs and sEVs. The results are presented as the average ± SD from three different spots on the chip (N = 1). B) Size of lEVs and sEVs as determined by NTA (N = 3).

Supplementary Figure 5. Proteins enriched in sEVs compared to lEVs. (A-B) The log2 fold change determined with quantitative proteomics for all annexins (A), and all heat shock proteins (B) quantified in the dataset. (C-D) The log2 fold change determined with quantitative proteomics for all integrins in EVs from all three cell lines (C), and in EVs from the BM and LN only (D). (E) The top 20 most enriched proteins in sEVs based on log2 fold change compared to lEVs. Light red: significant and fold change > 1 (log2 = 0); dark red: significant and fold change > 2 (log2 = 1) = enriched in sEVs. Light green: significant and fold change > -1 (log2 = 0); dark green: significant and fold change > -2 (log2 = -1) = enriched in lEVs. Grey: no significant enrichment in either sEVs or lEVs. Dotted lines on the Y-axis indicate log2 fold change = 1 and -1 (corresponding to fold change 2 and -2). Data presented as violin plots. N = 9 (N = 3 for each of the three cell lines).

Supplementary Figure 6. (A) Proteins enriched in lEVs compared to sEVs. (A) The log2 fold change determined with quantitative proteomics for proteins in our dataset with a log2 fold change above -1 that have previously been suggested to be enriched in the large EV subgroup, microvesicles/ectosomes compared to sEVs (26, 28, 29). (B) The log2 fold change determined with quantitative proteomics for proteins related to the proteins listed in Figure 7B and Supplementary Figure 6A that have previously been suggested to be upregulated in lEVs compared to sEVs.

Supplementary Figure 7. Nucleus-associated proteins are enriched in lEVs compared to sEVs. (A-B) The log2 fold change determined with quantitative proteomics for all CCR4-Not (A), and all nuclear pore complex (B) proteins quantified in the dataset. (C-D) The log2 fold change determined with quantitative proteomics for all heterogeneous nuclear ribonucleoproteins in EVs from all three cell lines (C), and in EVs from BM and LN only (D). Light red: significant and fold change > 1 (log2 = 0) = enriched in sEVs. Light green: significant and fold change > -1 (log2 = 0); dark green: significant and fold change > -2 (log2 = -1) = enriched in lEVs. Grey: no significant enrichment in either sEVs or lEVs. Dotted lines on the Y-axis indicate log2 fold change = 1 and -1 (corresponding to fold change 2 and -2). Data presented as violin plots. N = 9 (N = 3 for each of the three cell lines).

Supplementary Figure 8. Ribosomal proteins are enriched in lEVs compared to sEVs. (A-D) The log2 fold change determined with quantitative proteomics for all 60S (A), 40s (B), 39S (C) and 28S (D) ribosomal proteins quantified in the dataset. Light green: significant and fold change > -1 (log2 = 0); dark green: significant and fold change > -2 (log2 = -1) = enriched in lEVs. Grey: no significant enrichment in either sEVs or lEVs. Dotted lines on the Y-axis indicate log2 fold change = 1 and -1 (corresponding to fold change 2 and -2). Data presented as violin plots. N = 9 (N = 3 for each of the three cell lines).

Supplementary Table 1. 4851 proteins quantified in all lEVs and sEVs samples.
